# Income Related Inequality of Health Care Access in Japan: A Retrospective Cohort Study

**DOI:** 10.1371/journal.pone.0151690

**Published:** 2016-03-15

**Authors:** Misuzu Fujita, Yasunori Sato, Kengo Nagashima, Sho Takahashi, Akira Hata

**Affiliations:** 1 Chiba University Graduate School of Medicine, Department of Public Health, Chiba City, Chiba, Japan; 2 Chiba University Graduate School of Medicine, Department of Global Clinical Research, Chiba City, Chiba, Japan; 3 Chiba University Hospital, Clinical Research Center, Chiba City, Chiba, Japan; University of Florence, ITALY

## Abstract

The purpose of this retrospective cohort study was to analyze the association between income level and health care access in Japan. Data from a total of 222,259 subjects (age range, 0–74 years) who submitted National Health Insurance claims in Chiba City from April 2012 to March 2014 and who declared income for the tax period from January 1 to December 31, 2012 were integrated and analyzed. The generalized estimating equation, in which household was defined as a cluster, was used to evaluate the association between equivalent income and utilization and duration of hospitalization and outpatient care services. A significant positive linear association was observed between income level and outpatient visit rates among all age groups of both sexes; however, a significantly higher rate and longer period of hospitalization, and longer outpatient care, were observed among certain lower income subgroups. To control for decreased income due to hospitalization, subjects hospitalized during the previous year were excluded, and the data was then reanalyzed. Significant inverse associations remained in the hospitalization rate among 40–59-year-old men and 60–69-year-old women, and in duration of hospitalization among 40–59 and 60–69-year-olds of both sexes and 70–74-year-old women. These results suggest that low-income individuals in Japan have poorer access to outpatient care and more serious health conditions than their higher income counterparts.

## Introduction

There is ample evidence that lower socioeconomic status (SES), based on income level, education level, and occupation, is associated with higher all-cause mortality [1–5], cause-specific mortality such as deaths from cardiovascular disease, cancer, and injury [1, 3, 5, 6], and a decline in healthy life expectancy [4] in both Western countries and in Japan. However, the factors underlying these disparities have not been sufficiently investigated. Unhealthy lifestyle-related factors—including smoking [7–10], stress [10, 11], and physical inactivity [8–10] as well as poor utilization of health care resources, including regular check-ups [10] and cancer screening [12]—have been reported in individuals with low SES. Another factor could be difficulties in health care access. For example, uninsured individuals in the US, who typically have low SES, are less likely to obtain medical care after developing serious health problems caused by an unintentional injury or the onset of a chronic condition [13]. In addition, a study across 21 Organisation for Economic Co-operation and Development countries found that individuals with high income tend to see a specialist significantly more often than individuals with low income [14]. However, to the best of our knowledge, only four studies on the association between SES and health care utilization in Japan, a country that has a universal health care insurance system, have been conducted. One prospective cohort study comprising 1,024 individuals under 18 years of age reported finding no significant association between income level and health care utilization [15]. The other three studies were questionnaire surveys conducted on 2,060 people aged 20–89 years [16], 3,378 people aged 25–50 years [17], and 15,302 elderly people aged 65–100 years [18]. All three studies found disparities in health care access among low-income individuals. However, in these studies, diaries or self-reported questionnaires were used to collect data, and these methods have a serious drawback in that they are associated with inevitable selection and information biases due to low response rates and unreliable answers.

Japan has three major public health care insurance systems that provide universal coverage [19]. The Employees' Health Insurance system and the National Health Insurance (NHI) system are for individuals under 75 years of age; the former is for company or government employees and the latter for self-employed workers, farmers, retirees, or the unemployed. The Long Life Health Insurance system is for all elderly aged 75 or over. Generally, except for individuals receiving public assistance (*seikatsu-hogo*), a livelihood protection system that allows the destitute maintain a minimum standard of living and receive medical services at no cost, everyone living in Japan must be covered by a public health insurance system. The public health care insurance systems utilize an age-dependent benefit system. Basically, copayments are 20% of total medical expenses for beneficiaries less than 6 years of age, 30% for those between 7–69 years of age, and 10% for those 70 years of age or older. In addition, the maximum monthly copayment is capped based on income and age; for example, for individuals less than 70 years of age, copayments are capped at 35,000 yen for low-income individuals in a municipal-tax exempt household, 80,000 yen for middle-income individuals, and 150,000 yen for high-income with more than 6 million yen of annual household income. Furthermore, Chiba City implemented a medical fee reduction policy for all infants and children 15 years of age and under in FY 2013. Under this policy, the outpatient medical fee is 300 yen per visit for children aged 0–8 years and 500 yen for those aged 9–15 years. The inpatient medical fee is 500 yen per day for all children 15 years of age and under. In Japan, primary care in residential communities is generally provided by clinics. A patient is only referred to another institution when their local doctor judges that hospitalization is required. However, this system is not as strict as that in the United Kingdom as patients can freely access all medical institutions, albeit with some additional expenses.

In the current study, we aimed to examine the association between income level and health care access (hospitalization and outpatient visits) in a large retrospective cohort study comprising 222,259 Japanese subjects aged between 0–74 years. The advantage of this study is the accuracy of health care utilization and income data, the large sample size, and the wide range of age groups included.

## Materials and Methods

### Subjects

The subjects were 222,259 NHI beneficiaries (108,626 men, 113,633 women) in Chiba City. All subjects were registered from April 1, 2013 to March 31, 2014. Chiba is a large city located 40 km southeast of Tokyo with a population of 970,000. All the data used in this study were extracted from data obtained from Chiba City Hall, including income from tax records, residential area and household identifier number from a basic resident registry, and NHI claims.

### Ethics statement

To assure the anonymity of the subjects, before the data were provided to us, personal information—including names, addresses, and telephone numbers—were removed from the records, birth dates were uniformly converted into the first day of each month, and unique identifier numbers for personal, household, and health insurance claims were converted into random numbers at Chiba City Hall. Because this was an observational study using existing data administrated by Chiba City Hall, consent from each subject was deemed unnecessary. The Research Ethics Committee of the Graduate School of Medicine, Chiba University, approved this study. The study was conducted in accordance with the Declaration of Helsinki.

### Main predictor

The main predictor was equivalent household income. Individual annual income, which included business, real estate, interest, dividends, employment, and retirement, and other income, declared to Chiba City Hall for the tax period from January 1 to December 31, 2012 was used. Self-employed workers whose annual net income was less than 380,000 yen are exempted from income tax and not required to declare in Japan. We considered the income of those who did not declare any income as 0 yen. The number of people per household was obtained by counting the persons with the same household number. Family members per household in this study included NHI beneficiaries and the head of household, regardless of whether he/she was a NIH beneficiary. Household income was calculated by adding net income of the household members, as mentioned above. Equivalent household income was calculated as follows: household income divided by the square root of the number of household members [20]. We divided the subjects into five categories based on equivalent income (0.00, 0.01–1.00, 1.01–2.00, 2.01–3.00, and 3.01 or more [million yen]), and into three categories based on the number of family members (1 or 2, 3, and 4 or more).

### Insurance claims data and outcomes

Chiba City NHI claim data, including cost, number of days, and primary diagnosis according to the Table of International Classification of Disease for the Use of Social Insurance established by Ministry of Health, Labour and Welfare in Japan [21], submitted to the Federation of NHI Associations in Chiba Prefecture from April 2012 to March 2014 (fiscal year [FY] 2012 and 2013) were obtained. Outcomes were the utilization and the duration of hospitalization and outpatient care based on health insurance claims during one year. The total number of the claims submitted during this period was 8,163,241. Of these claims, 18,505 (0.2%) were excluded due to incomplete records. The remaining 8,144,736 claims comprised 4,121,739 outpatient claims, 91,170 hospitalization claims, and 3,931,827 dental care, pharmacy, and other claims, including home-visit nursing and *judo* therapy. Outpatient claims included both primary care and specialist services, but not preventive care such as annual health checkups. Hospitalization claims included all medical care during hospitalization except for childbirth. We determined the precise utilization and the duration of hospitalization and outpatient care over one year using the claims submitted in FY 2013. Binary variables (0 = no, 1 = yes) were used for utilization of hospitalization and outpatient care. The duration of hospitalization and outpatient care was calculated by adding the days described in claims and used as count data. The FY 2012 claims were used to exclude the subjects hospitalized in FY 2012 in a sensitivity analysis.

### Additional data

We divided the subjects into five age groups (0–15, 16–39, 40–59, 60–69, and 70–74 years of age). However, due to the comparatively small number of hospitalized subjects aged 0–15 years, especially those categorized as being in the highest income group (40 boys, 19 girls), the 0–15 and 16–39 year age groups were combined for analysis. We also obtained data in five categories based on residential area (Chuo, Hanamigawa, Inage, Wakaba, Midori, and Mihama) in April 2, 2013.

### Statistical analysis

For continuous variables, the mean and standard deviation were calculated, and analysis of variance and a linear regression model were used to perform the trend test in univariate analysis. The χ^2^ and Cochran-Armitage tests were performed for categorical variables.

The generalized estimating equation (GEE) was used to evaluate the association between equivalent income and utilization and duration of hospitalization and outpatient care services [22]. We treated each household as a cluster. The utilization data were binary variables, and the duration of hospitalization and outpatient care were count data. Therefore, we used a binomial distribution with a logit link function to analyze utilization, and a Poisson distribution with a log link function to analyze duration. In the analysis of the duration of hospitalization and outpatient care, the subjects were limited to those who received outpatient care services or were hospitalized during the survey year (i.e., utilization = 1). Since we considered that the association between income level and health care access might be different based on sex and age, we evaluated the association using the following five models: Model 1 for main associations (sex, age, and equivalent income); Model 2 for main associations with two-way interaction (sex*equivalent income); Model 3 for main associations with two-way interaction (age*equivalent income); Model 4 for main associations with two-way interactions (sex*equivalent income and age*equivalent income); and Model 5 for main associations, two-way interactions (sex*age, sex*equivalent income, and age*equivalent income), and three-way interaction (sex*age*equivalent income), adjusting for residential area and the number of household members. We adjusted for residential area because differences were found between areas in resources for medical services. For example, the numbers of medical facilities per 100,000 people in Chuo, Midori, Mihama, Inage, Hanamigawa, and Wakaba were 88.3, 73.8, 61.9, 58.3, 55.3, and 51.5, respectively [23]. Model selection was decided according to quasi-likelihood under the independence model criterion [24]. Probabilities of utilization and the duration of hospitalization and outpatient care services during one year, as predicted by the GEE, were calculated, and a linear trends test for equivalent income was performed. In addition, in a sensitivity analysis to exclude the subjects whose income might have decreased due to hospitalization, the association between equivalent income and utilization and duration of hospitalization was investigated in the restricted subjects who had been beneficiaries of NHI in Chiba City and had not been hospitalized in the previous year (FY 2012). Furthermore, we performed additional analysis to identify the primary diagnosis for hospitalization in each income category. First, we extracted hospitalization claims for FY 2013, and then we performed a cross-tabulation of income groups and primary diagnoses for each age and sex group. We performed the same analysis using hospitalization claims for FY 2013 after excluding claims for the subjects hospitalized in FY 2012.

Results were considered significant when the two-tailed *P* value was < 0.05. All statistical analyses were performed using SAS software version 9.4 (SAS Institute, Cary, NC).

## Results

The results of univariate analysis of the subjects’ characteristics and health care access among equivalent income groups are shown in Table 1. Significant differences were found in all characteristics, utilizations, and durations of hospitalization and outpatient care services among all income levels. The subjects with lower equivalent incomes were less likely to utilize outpatient care, but more likely to be hospitalized (both *P* values for linear trends < 0.001). In addition, the duration of hospitalization and outpatient care was greater among those with lower incomes (both *P* values for linear trends < 0.001).

**Table 1:** Subjects’ characteristics and the utilization and duration of hospitalization and outpatient care.

		Equivalent income (million yen)						
	All	0.00	0.01–1.00	1.01–2.00	2.01–3.00	≥3.01	*P*-value	*P*-value for trends
Number of subjects	222,259	38,108	70,710	67,000	24,435	22,006		
Men (%)^a^	108,626 (48.9)	16,819 (44.1)	33,340 (47.2)	34,182 (51.0)	13,263 (54.3)	11,022 (50.1)	<0.001	<0.001
sAge^b^	52.5 (19.9)	50.7 (19.5)	53.6 (20.3)	53.8 (20.0)	51.3 (19.5)	49.3 (18.8)	<0.001	<0.001
Number of family members^a^								
1 or 2 (%)	155,835 (70.1)	32,310 (84.8)	48,495 (68.6)	45,216 (67.5)	15,822 (64.8)	13,992 (62.6)	<0.001	
3 (%)	37,338 (16.8)	3,440 (9.0)	13,160 (18.6)	11,840 (17.7)	4,815 (19.7)	4,082 (18.6)		
4 or more (%)	29,086 (13.1)	2,358 (6.2)	9,054 (12.8)	9,944 (14.8)	3,798 (15.5)	3,932 (17.9)		
Residence area^a^								
Chuo (%)	44,382 (20.0)	8,686 (22.8)	13,791 (19.5)	12,423 (18.5)	4,912 (20.1)	4,570 (20.8)	<0.001	
Hanamigawa (%)	41,784 (18.8)	6,980 (18.3)	13,463 (19.0)	12,744 (19.0)	4,511 (18.5)	4,086 (18.6)		
Inage (%)	35,548 (16.0)	5,871 (15.4)	11,177 (15.8)	10,861 (16.2)	3,856 (15.8)	3,783 (17.2)		
Wakaba (%)	42,236 (19.0)	7,892 (20.7)	13,043 (18.5)	12,756 (19.0)	4,701(19.2)	3,844 (17.5)		
Midori (%)	26,128 (11.8)	3,828 (10.1)	8,451 (12.0)	8,199 (12.2)	2,990 (12.2)	2,660 (12.1)		
Mihama (%)	32,181 (14.5)	4,851 (12.7)	10,785 (15.3)	10,017 (15.0)	3,465(14.2)	3,063 (13.9)		
Utilization of outpatient care^a^	180,256 (81.1)	28,058 (73.6)	57,902 (81.9)	55,923 (83.5)	19,936 (81.6)	18,437 (83.8)	<0.001	<0.001
Utilization of hospitalization^a^	15,425 (6.9)	2,996 (7.9)	5,184 (7.3)	4,519 (6.7)	1,462 (6.0)	1,264 (5.7)	<0.001	<0.001
Duration of outpatient care^b^^,^^c^	15.8 (20.9)	16.9 (23.6)	16.5 (22.1)	15.9 (20.2)	14.4 (18.7)	13.4 (16.8)	<0.001	<0.001
Duration of hospitalization^b^^,^ ^d^	33.2 (72.5)	65.0 (115)	30.2 (61.5)	23.5 (51.8)	19.1 (43.2)	21.0 (49.9)	<0.001	<0.001

^a^Data are expressed as number of subjects (percent). The χ^2^ and Cochran-Armitage tests were used.

^b^Data are expressed as mean (standard deviation). Analysis of variance and a linear regression model were used to perform the trend test.

^c^Subjects are those who utilized outpatient care during the survey year (n = 180,256).

^d^Subjects are those hospitalized during the survey year (n = 15,425).

As of June 30, 2015, 1 USD was the equivalent of 122.72 JPY.

### Model selection

Coefficients estimated by the GEE and quasi-likelihood under the independence model criterion for utilization of outpatient care services and hospitalization in all subjects are shown in S1 and S2 Tables, respectively. The duration of care among 180,256 subjects who received outpatient care services during the survey year is shown in S3 Table, and the duration of hospitalization among 15,425 subjects who were hospitalized during the survey year is shown in S4 Table. Model 5 had the best goodness of fit for all outcomes, and therefore the results from this model are described as follows.

### Utilization of hospitalization and outpatient care services

**Fig 1:**
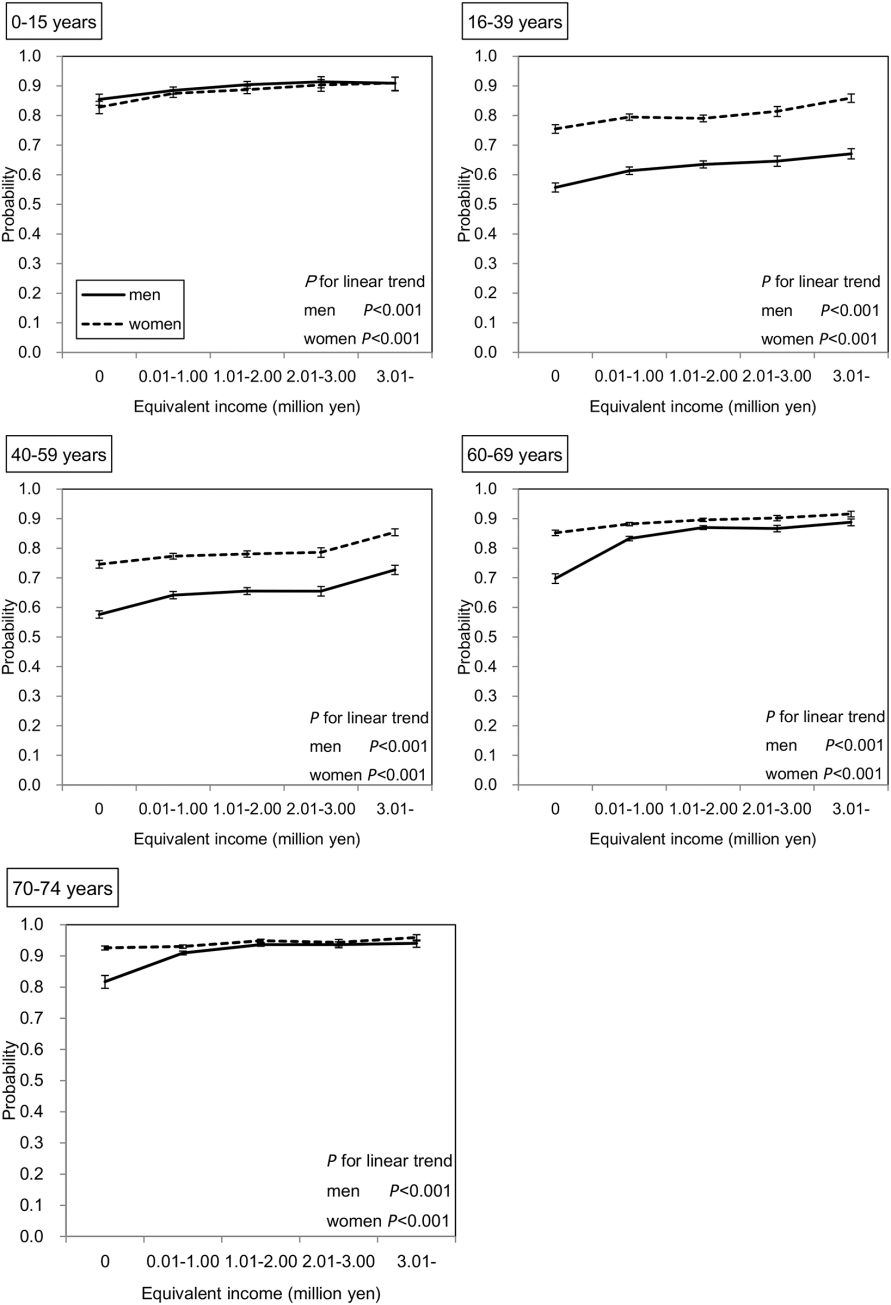
Association between equivalent income and outpatient utilization rate in all subjects. Adjusted means and 95% confidence intervals (CIs) are shown. The number of subjects was 222,259. The probabilities were predicted by the Generalized Estimating Equation (GEE) with binomial distribution and logit function considering the correlation within households. This model has main associations (age, sex, and equivalent income), two-way interactions (age*sex, age*equivalent income, and sex*equivalent income), three-way interaction (age*sex*equivalent income), and two covariates (the number of family members and residence).

The probabilities of utilization of outpatient care services for one year as predicted by the GEE are shown in Fig 1. In all groups of both sexes, the subjects with lower income were less likely to utilize outpatient care services (all *P* values for linear trends < 0.001). When linear trends were compared between income and utilization of outpatient care in the 0–15 years age group, who are covered by the medical fee reduction policy, with other age groups, significant differences were observed in only two pairs (0–15 years vs. 60–69 years, and 0–15 years vs. 70–79 years in men [p<0.001 for both]).

**Fig 2:**
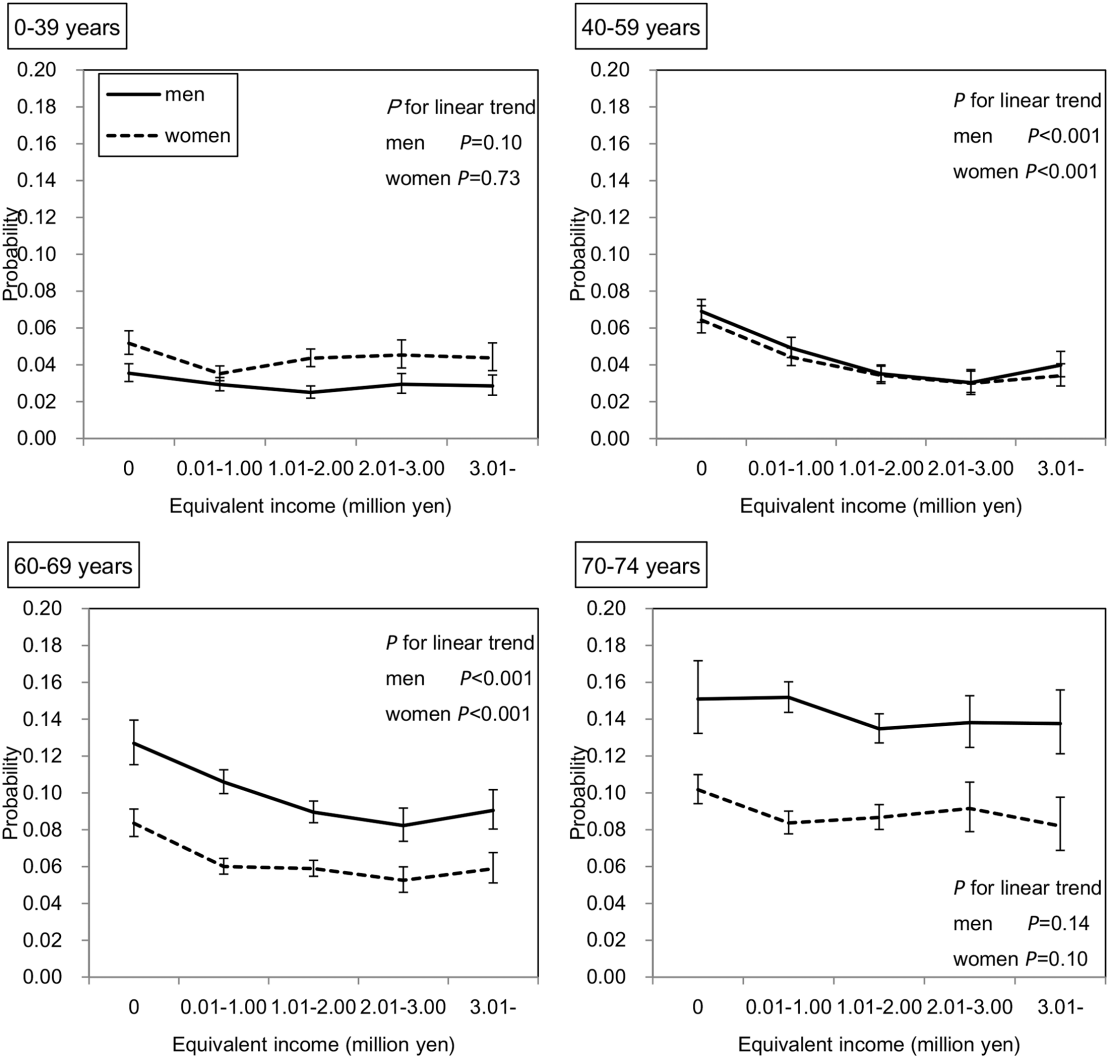
Association between equivalent income and hospitalization rate in all subjects. Adjusted means and 95% CIs are shown. The number of subjects was 222,259. The probabilities were predicted by the GEE with binomial distribution and logit function considering the correlation within households. This model has main associations (age, sex, and equivalent income), two-way interactions (age*sex, age*equivalent income, sex*equivalent income), three-way interaction (age*sex*equivalent income), and two covariates (number of family members and residence).

**Fig 3:**
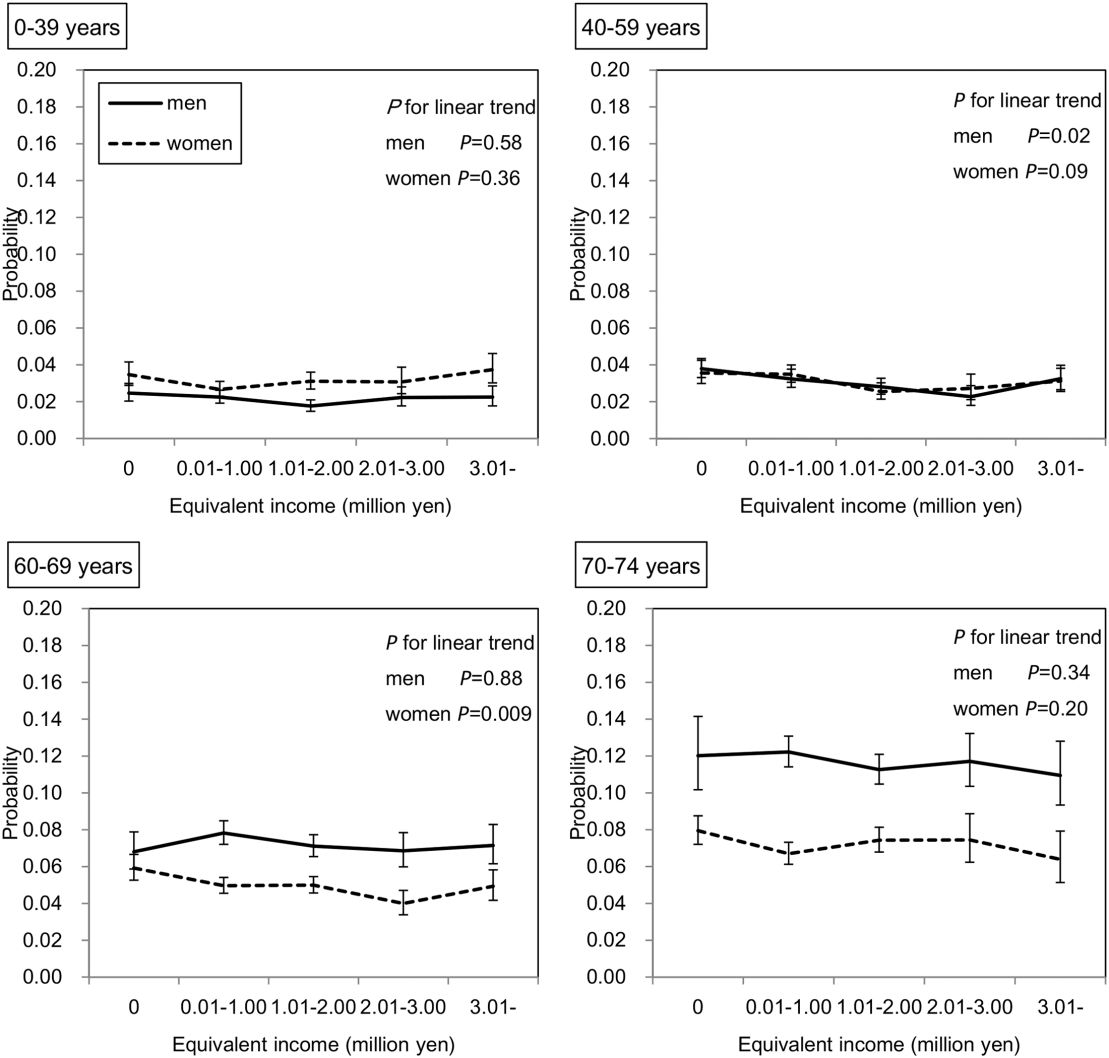
Association between equivalent income and hospitalization rate in the limited subjects. Adjusted means and 95% CIs are shown. The subjects were 183,681 National Health Insurance (NHI) beneficiaries in Chiba City who had not been hospitalized in the previous year (FY 2012). The probabilities were predicted by the GEE with binomial distribution and logit function considering the correlation within household. This model has main associations (age, sex, and equivalent income), two-way interactions (age*sex, age*equivalent income, sex*equivalent income), three-way interaction (age*sex*equivalent income), and two covariates (the number of family members and residence).

On the other hand, as shown in Fig 2, significant inverse linear associations were found between equivalent income and probability of hospitalization in subjects of both sexes aged 40–59 years and 60–69 years (all *P* values for linear trends < 0.001). As shown in Fig 3, when the subjects were limited to those who had not been hospitalized in the previous year, these significant inverse associations remained in men aged 40–59 years (*P* value for linear trend = 0.02) and in women aged 60–69 years (*P* value for linear trend = 0.009).

### Duration of hospitalization and outpatient care

**Fig 4:**
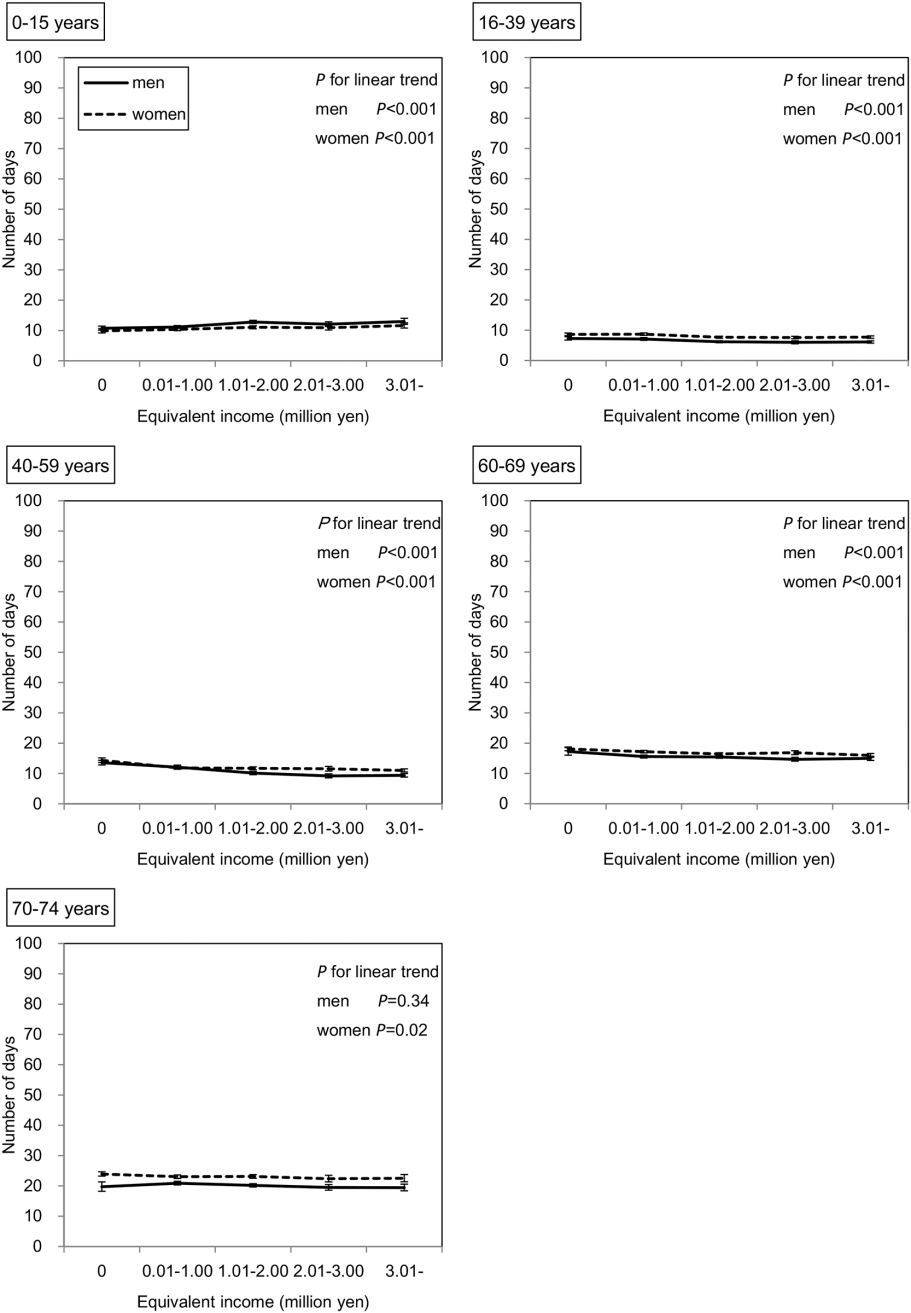
Association between equivalent income and duration of outpatient care. Adjusted means and 95% CIs are shown. The number of subjects who received outpatient care services in the survey year was 180,256. The duration of outpatient care was predicted by the GEE with a Poisson distribution and log function considering the correlation within household. This model has main associations (age, sex, and equivalent income), two-way interactions (age*sex, age*equivalent income, sex*equivalent income), three-way interaction (age*sex*equivalent income), and two covariates (the number of family members and residence).

**Fig 5:**
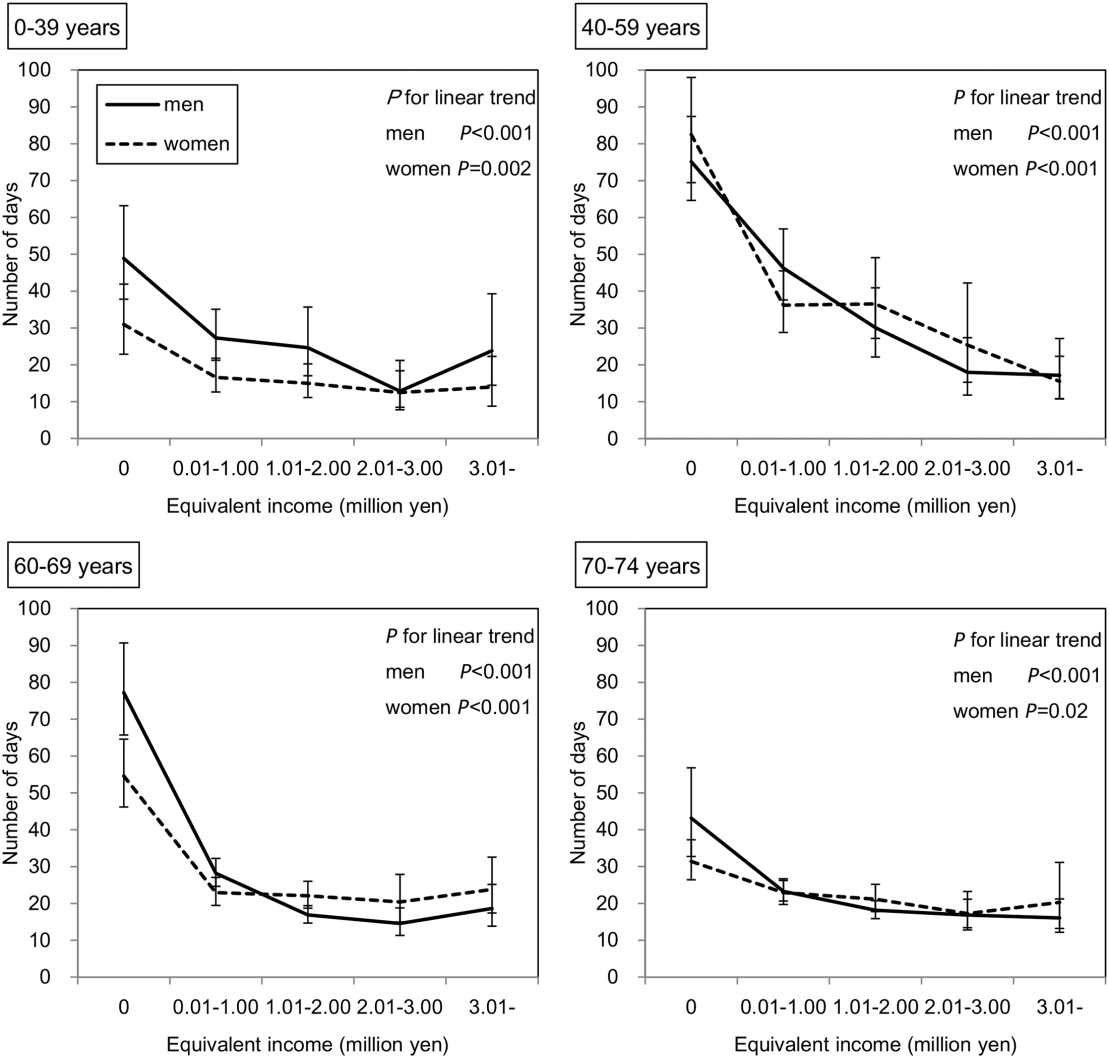
Association between equivalent income and duration of hospitalization. Adjusted means and 95% CIs are shown. The number of subjects who were hospitalized in the survey year was 15,425. The duration of hospitalization was predicted by the GEE with a Poisson distribution and log function considering the correlation within household. This model has main associations (age, sex, and equivalent income), two-way interactions (age*sex, age*equivalent income, sex*equivalent income), three-way interaction (age*sex*equivalent income), and two covariates (the number of family members and residence).

**Fig 6:**
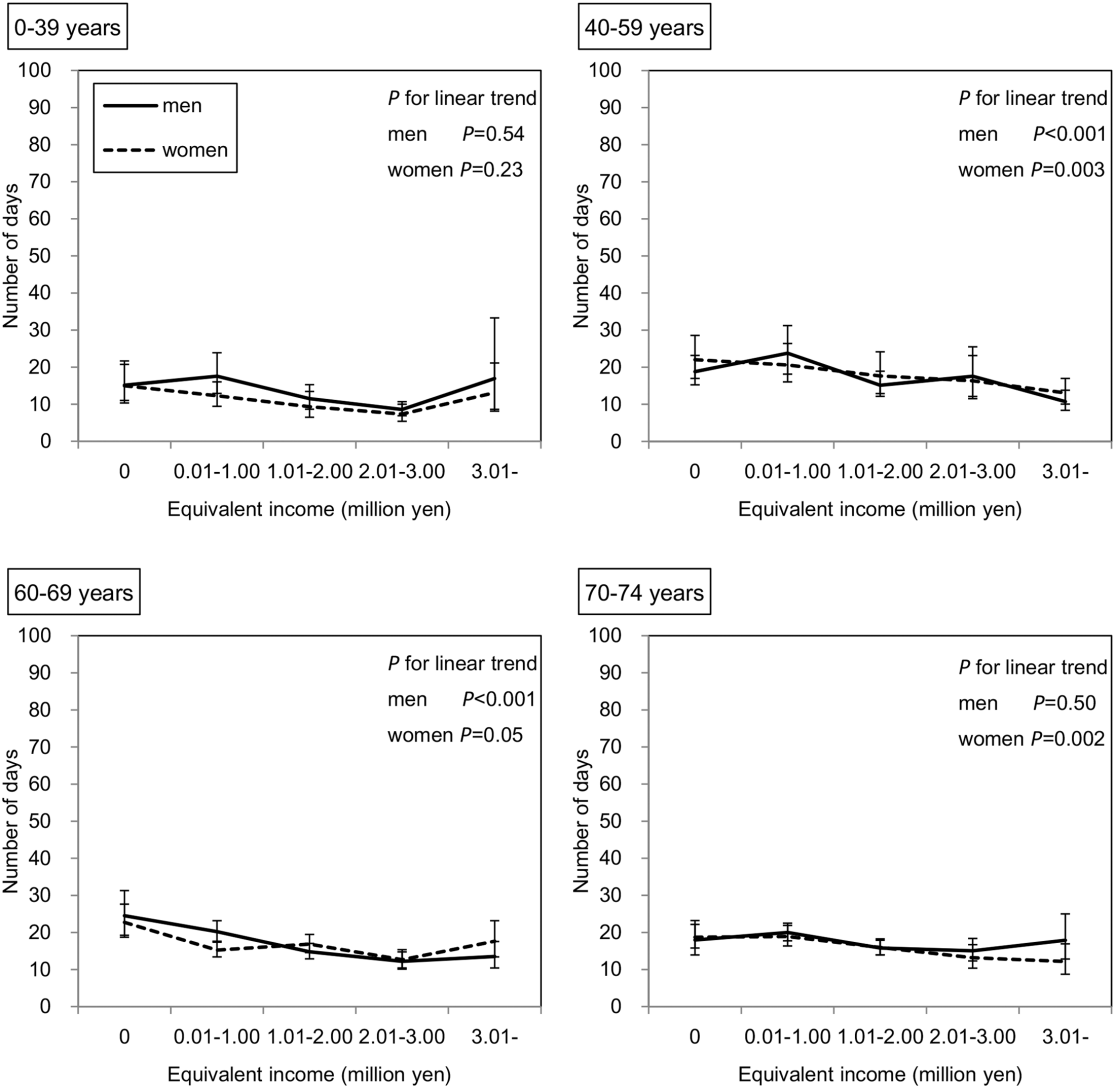
Association between equivalent income and duration of hospitalization in the limited subjects. Adjusted means and 95% CIs are shown. The number of subjects who received hospitalization in survey year, had been beneficiaries of NHI in Chiba City, and had not been hospitalized in the previous year (FY 2012) was 9,813. The duration of hospitalization was predicted by the GEE with a Poisson distribution and log function considering the correlation within household. This model has main associations (age, sex, and equivalent income), two-way interactions (age*sex, age*equivalent income, sex*equivalent income), three-way interaction (age*sex*equivalent income), and two covariates (the number of family members and residence).

Among 180,256 subjects who received outpatient care in the survey year, the duration of outpatient care during one year as predicted by the GEE is shown in Fig 4. In the subjects of both sexes aged 0–15 years, those with lower incomes had a significantly lower duration of outpatient care (both *P* values < 0.001). In contrast, in the other age groups, except for men aged 70–74 years, the duration of outpatient care was significantly higher among those with lower incomes. Similarly, as shown in Fig 5, among 15,425 subjects who were hospitalized in the survey year, those with lower incomes in all age groups of both sexes had a significantly greater duration of hospitalization; however, this inverse association seemed to be stronger for hospitalization than for outpatient care. For example, in men aged 40–59 years, the adjusted mean durations of outpatient care were 13.6 and 9.4 days in the lowest and highest income groups, respectively, and the adjusted mean durations of hospitalization were 77.2 and 18.7 days, respectively. As shown in Fig 6, when the subjects were restricted to those who had not been hospitalized in the previous year, these inverse associations remained in both sexes aged 40–59 and 60–69 years and in women aged 70–74 years.

### Primary diagnosis for hospitalization in each income category

**Fig 7:**
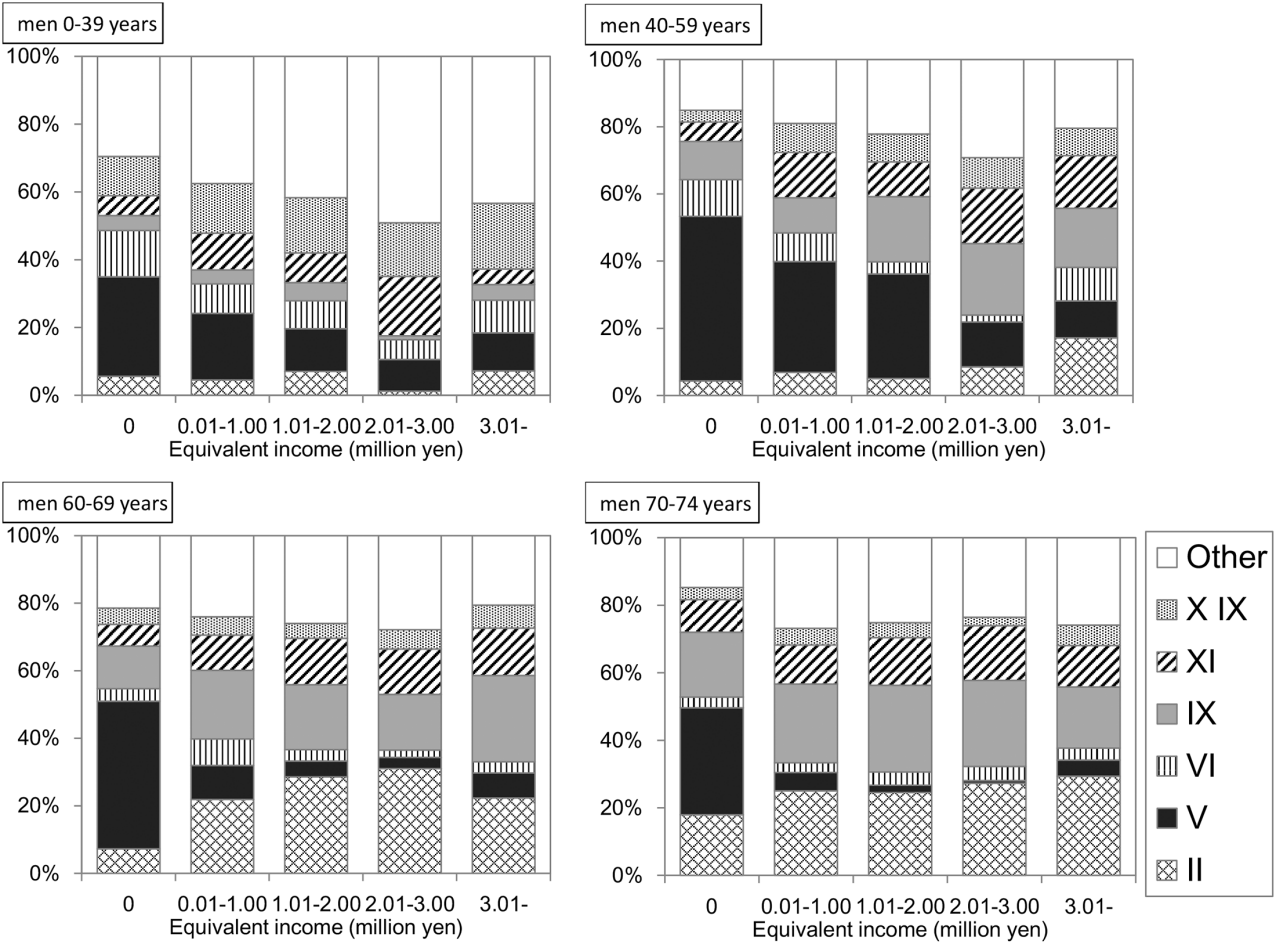
Primary diagnosis for hospitalization in men. The proportions of the claims with each diagnosis to the total claims for hospitalization are shown. The number of claims in this tabulation was 19,481. II: Neoplasms. V: Mental and behavioral disorders. VI: Disease of the nervous system. IX: Disease of the circulatory system. XI: Disease of the digestive system. XIX: Injury, poisoning and certain other consequences of external causes.

**Fig 8:**
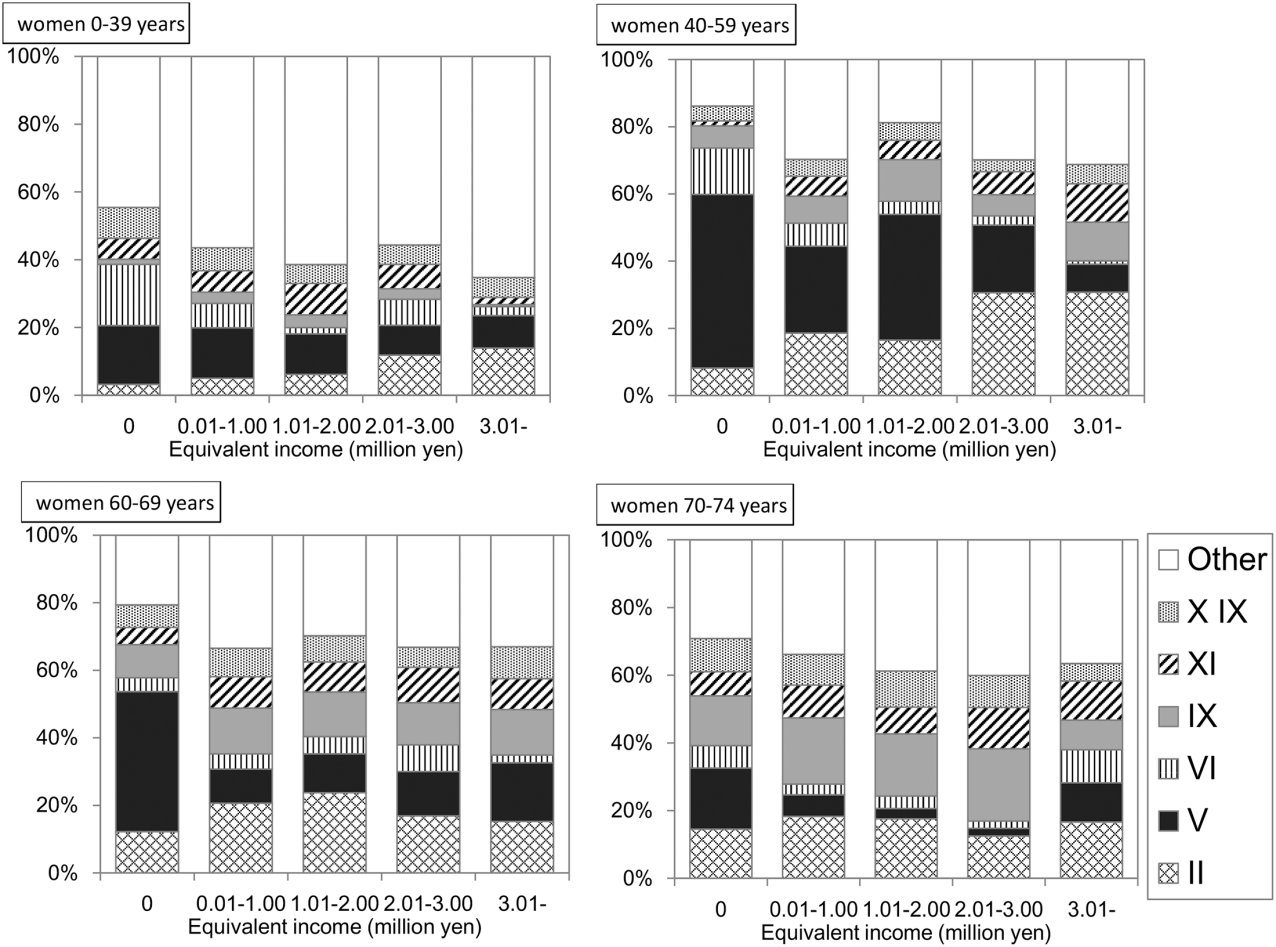
Primary diagnosis for hospitalization in women. The proportions of the claims with each diagnosis to the total claims for hospitalization are shown. The number of claims in this tabulation was 15,263. II: Neoplasms. V: Mental and behavioral disorders. VI: Disease of the nervous system. IX: Disease of the circulatory system. XI: Disease of the digestive system. XIX: Injury, poisoning and certain other consequences of external causes.

A total of 34,964 hospitalization claims were derived from 15,425 hospitalized individuals in FY 2013. After excluding 220 claims (0.63%) due to a lack of a primary diagnosis for hospitalization, a total of 34,744 claims (19,481 for men, 15,263 for women) were cross-tabulated using income and primary diagnoses. As shown in Figs 7 and 8, the proportion of claims for “mental and behavioral disorders” was remarkable among the lower income individuals across all age groups and sex. However, in the subjects who had not been hospitalized during the previous year, this proportion appeared to decrease (S1 and S2 Figs).

## Discussion

The findings of this study revealed that the utilization rate of outpatient care services was significantly lower in subjects in all age groups of both sexes with lower incomes, suggesting that individuals with lower SES in Japan make fewer outpatient visits. On the other hand, significantly higher hospitalization rates were associated with lower income in subjects of both sexes aged 40–59 and 60–69 years. In addition, those with lower incomes had significantly longer durations of hospitalization and outpatient care. These results suggest that individuals with lower incomes are more likely to develop severe diseases than those with higher incomes.

In previous studies using data from methods known to have inevitable selection and information biases such as diaries or self-reported questionnaires with low response rates [16–18], lower levels of health care access among individuals with low SES in Japan have also been found. Hirai et al. [4] reported that the overall response rate for self-reported mail survey questionnaires, especially responses for items on income, were significantly lower in subjects with lower incomes. They also reported that, when self-administered mail survey methods were employed, inequalities in health between individuals with different SES levels tend to be underestimated due to missing data. We used low biased income and health care access data; therefore, the present study has a definitive advantage over previous studies.

It is noteworthy that this is the first study to find inequality in health care access among younger subjects (aged 0–15 years) in Japan. No association between health care access and annual family income was reported in a previous prospective cohort study in Japan comprising subjects aged 0–18 years [15]. Many municipalities in Japan have implemented a medical fee reduction policy for infants and children. The subjects of the program in Chiba City in FY 2013 were citizens aged 0–15 years who had outpatient medical fees of either 300 yen (0–8 years) or 500 yen (9–15 years) per visit. Despite this policy, a positive association was found between equivalent income and utilization of outpatient care services in this age group. Furthermore, this study showed that the linear trend between income and utilization of outpatient care in the 0–15 years age group was not significantly different compared with that in most other age groups. We did not find that the medical fee reduction policy had a definite effect on eliminating the inequality of health care access. Similarly, although those aged 70 years or older are charged 10% of their total medical fees, compared with 30% in other age groups, significantly lower outpatient utilization rates were observed among those with lower incomes. Therefore, even with reduced payments, those with lower incomes may still feel an excessive cost burden. Factors other than cost,—such as lower levels of functional health literacy [25], less knowledge about disease [26], and less free time [27] that are found more frequently among individuals with lower SES—might explain this association.

A higher hospitalization rate was also found in lower income subjects aged 40–59 and 60–69 years in both sexes; this result supports those found in previous studies [17, 28]. Furthermore, we found that the durations of outpatient care in those aged 16–39, 40–59, and 60–69 years in both sexes, and in women aged 70–74 years, and of hospitalization in all age groups, were greater in those with lower incomes. These results suggest that individuals with lower incomes tend to develop more severe diseases than those with higher incomes. However, there are two possible explanations for this inverse association. One is that low income is a consequence of hospitalization. The other is that the situation of low income in itself somehow increases the tendency for hospitalization. In order to evaluate the former, the association between hospitalization and subsequent income needs to be investigated. Although we did not have sufficient data to perform this analysis in the present study, we did find a significant inverse association between income level and hospitalization rate in middle aged subjects, who are typically regarded as the primarily earners. In addition, the association between income and duration of hospitalization seemed to be stronger that for income and duration of outpatient care. This may indicate that long-term hospitalization leads to a reduced income. Based on these results, low income seems to be a result of hospitalization. On the other hand, for the latter possibility, when subjects were limited to those who had not been hospitalized during the previous year, in other words, when the association was assessed between income in the previous year and the new rate of hospitalization during one year, significant inverse associations remained in some age groups. Therefore, low income was not only the result of hospitalization, but also the cause of a higher hospitalization rate and a longer duration of hospitalization to some extent.

In the present study, extreme long-term hospitalization was noted in the subjects with lower incomes. For example, among men aged 40–59 years, the adjusted mean duration of hospitalization was 77.2 days in the lowest income group. To find a possible explanation for this, the primary reason for hospitalization was assessed for each income category. The proportion of those hospitalized for “mental and behavioral disorders” was greater among both men and women with lower incomes. According to a survey by the Ministry of Health, Labour and Welfare [29], the duration of hospitalization for “mental and behavioral disorders” per claim is 28.0 days, which is longer than that of other diseases such as “neoplasms” (12.6 days), “diseases of the circulatory system” (15.0 days), and “Injury, poisoning and certain other consequences of external causes” (14.7 days). A greater number of patients with lower incomes hospitalized for “mental and behavioral disorders” would reflect a longer duration of hospitalization in this group. In sensitivity analysis, when subjects were restricted to those not hospitalized during the previous year, the duration for hospitalization in the lowest income group decreased. Similarly, among these subjects, the proportion of claims with “mental and behavioral disorders” also decreased (S1 and S2 Figs).

The less frequent use of outpatient care and the higher frequency of hospitalization among lower income individuals in our study population suggested that poor access to outpatient care leads to higher hospitalization rates among lower income individuals. Although quite a number of previous studies have reported that hospitalization for ambulatory care-sensitive conditions (ACSCs), which is avoidable with successful management in the community, is more prevalent among low-income individuals than their high-income counterparts [30–32] no consensus has been reached on the role outpatient care utilization plays in this phenomenon. Trachtenberg et al. claimed that the income-based disparity in hospitalization for ACSCs cannot be explained by factors directly related to outpatient care utilization [32]. In this study, we analyzed hospitalization as a whole, not specifically for ACSCs, and because the association between income and consequent hospitalization before and after adjusting for outpatient care utilization was not feasible with the obtained data, we could not determine the effect of the primary care as a mediator between lower income and higher hospitalization for ACSCs. Further studies are needed to clarify this issue.

Our study did have some limitations. A previous study evaluating inequalities in health care access according to income in 21 Organisation for Economic Co-operation and Development countries was performed by adjusting detailed health-related factors such as the presence and degree of chronic physical impairment, mental health problems, illnesses, or disabilities [14]. Due to the nature of our study design, data regarding health-related factors were limited. It is well known that people with low SES tend to have more health problems than those with high SES. For example, individuals with low SES have a higher prevalence of diabetes [33, 34], hypertension [35], and chronic kidney disease [36]. Therefore, if the availability of outpatient care services were equal between income levels, individuals with low incomes would utilize such services more frequently than those high incomes, which would be the opposite result to what we found in the present study. This suggests that individuals in Japan with low incomes in all age groups of both sexes tend to restrict their outpatient visits. Second, the subjects in this study were all beneficiaries of NHI in Chiba City, and therefore the generalizability of our results is limited. The health care system and medical policy varies from country to country. In Japan, beneficiaries can receive medical care while paying only 10–30% of medical fees, and the maximum monthly copayment is capped based on income and age. Thus, in Japan, people with low income and serious diseases would find it comparatively easy to access hospitalization given these health care systems. The results of this study might therefore not be applicable to the situation in other countries. Furthermore, there is limited generalizability even in for other Japanese contexts. For example, this sample did not include individuals who received public assistance (*seikatsu-hogo*). Matsuyama et al. [37] reported that low-income respondents were not as likely to use dental prostheses, but the poorest respondents, whose household income was < $5,000, had a dental prosthesis utilization rate comparable to the highest income group in Japan. One possible reason for this reverse phenomenon was thought to be the free access to medication available to those who receive public assistance [37]. In contrast, in our study, the poorest people had the lowest probability of utilizing outpatient care services in all age groups of both sexes. The eligibility criteria for receiving public assistance depend on not only household income, but also living conditions such as the presence of family members who do not live together or have the ability to work. Therefore, in some cases, people could not receive public assistance, even though they reported no household income. In Japan, these individuals would be NHI beneficiaries. Because the subjects in our study did not include those individuals receiving public assistance, the poorest people still paid 10–30% of their medical fees. This may explain why no reverse patterns of social inequality were observed among the poorest people in our study. In addition, our study did not include company or government employees. Occupation has been shown to be an important determinant of health care utilization [38, 39]. Therefore, studies on health care access among health insurance beneficiaries who are either employed or receiving public assistance are also warranted.

### Conclusions

Based on data from reported income and medical insurance claims from a local government institution, we found inequalities in access to outpatient care among income levels in Japan. However, we also found a higher hospitalization rate and a longer duration of hospitalization and outpatient care among individuals with lower incomes. Based on these results, individuals with lower incomes appear have a greater tendency to develop more serious diseases than those with higher incomes.

## Supporting Information

S1 Fig Primary diagnosis for hospitalization in men who had not been hospitalized during the previous year. The proportion of claims with each diagnosis to the total number of hospitalization claims is shown. The number of claims in this tabulation was 10,648. II: Neoplasms. V: Mental and behavioral disorders. VI: Disease of the nervous system. IX: Disease of the circulatory system. XI: Disease of the digestive system. XIX: Injury, poisoning and certain other consequences of external causes.
(TIF)

S2 Fig Primary diagnosis for hospitalization in women who had not been hospitalized during the previous year. The proportion of claims with each diagnosis to the total number of hospitalization claims is shown. The number of claims in this tabulation was 8,911. II: Neoplasms. V: Mental and behavioral disorders. VI: Disease of the nervous system. IX: Disease of the circulatory system. XI: Disease of the digestive system. XIX: Injury, poisoning and certain other consequences of external causes.
(TIF)

S1 Table Coefficients estimated by the generalized estimating equation for the association between equivalent income and utilization of outpatient care services. Abbreviations: CI, confidence interval; QIC, quasi-likelihood under the independence model criterion. ^a^Age is expressed as years. ^b^Equivalent income is expressed as million yen. The number of subjects was 222,259. Binominal distribution and logit link function were defined in this model.
(DOCX)

S2 Table Coefficients estimated by the generalized estimating equation for the association between equivalent income and hospitalization. Abbreviations: CI, confidence interval; QIC, quasi-likelihood under the independence model criterion. ^a^Age is expressed as years. ^b^Equivalent income is expressed as million yen. The number of subjects was 222,259. Binominal distribution and logit link function were defined in this model.
(DOCX)

S3 Table Coefficients estimated by the generalized estimating equation for the association between equivalent income and duration of outpatient care. Abbreviations: CI, confidence interval; QIC, quasi-likelihood under the independence model criterion. ^a^Age is expressed as years. ^b^Equivalent income is expressed as million yen. The number of subjects who received outpatient care services in survey year was 180,256. Poisson distribution and log link function were defined in this model.
(DOCX)

S4 Table Coefficients estimated by the generalized estimating equation for the association between equivalent income and duration of hospitalization. Abbreviations: CI, confidence interval; QIC, quasi-likelihood under the independence model criterion. ^a^Age is expressed as years. ^b^Equivalent income is expressed as million yen. The number of subjects who were hospitalized in the survey year was 15,425. Poisson distribution and log link function were defined in this model.
(DOCX)
