# Supplementary figures and images for: Income Related Inequality of Health Care Access in Japan: A Retrospective Cohort Study
Source: PLoS One. 2016 Mar 15;11(3):e0151690. doi: 10.1371/journal.pone.0151690 (PMC4792389; doi:10.1371/journal.pone.0151690)

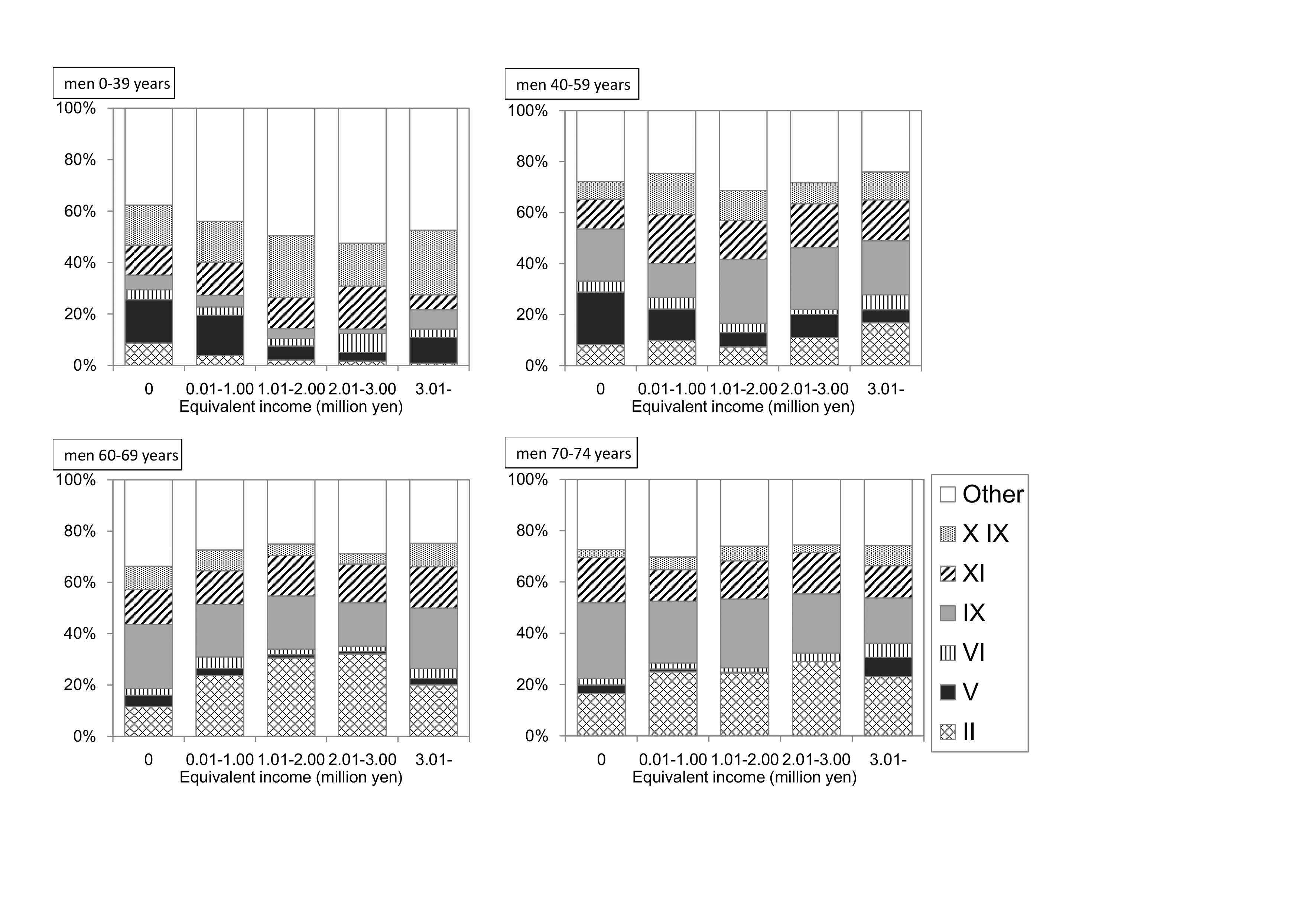

Supplement: S1 Fig — The proportion of claims with each diagnosis to the total number of hospitalization claims is shown. The number of claims in this tabulation was 10,648. II: Neoplasms. V: Mental and behavioral disorders. VI: Disease of the nervous system. IX: Disease of the circulatory system. XI: Disease of the digestive system. XIX: Injury, poisoning and certain other consequences of external causes. (TIF) [file pone.0151690.s001.tif]

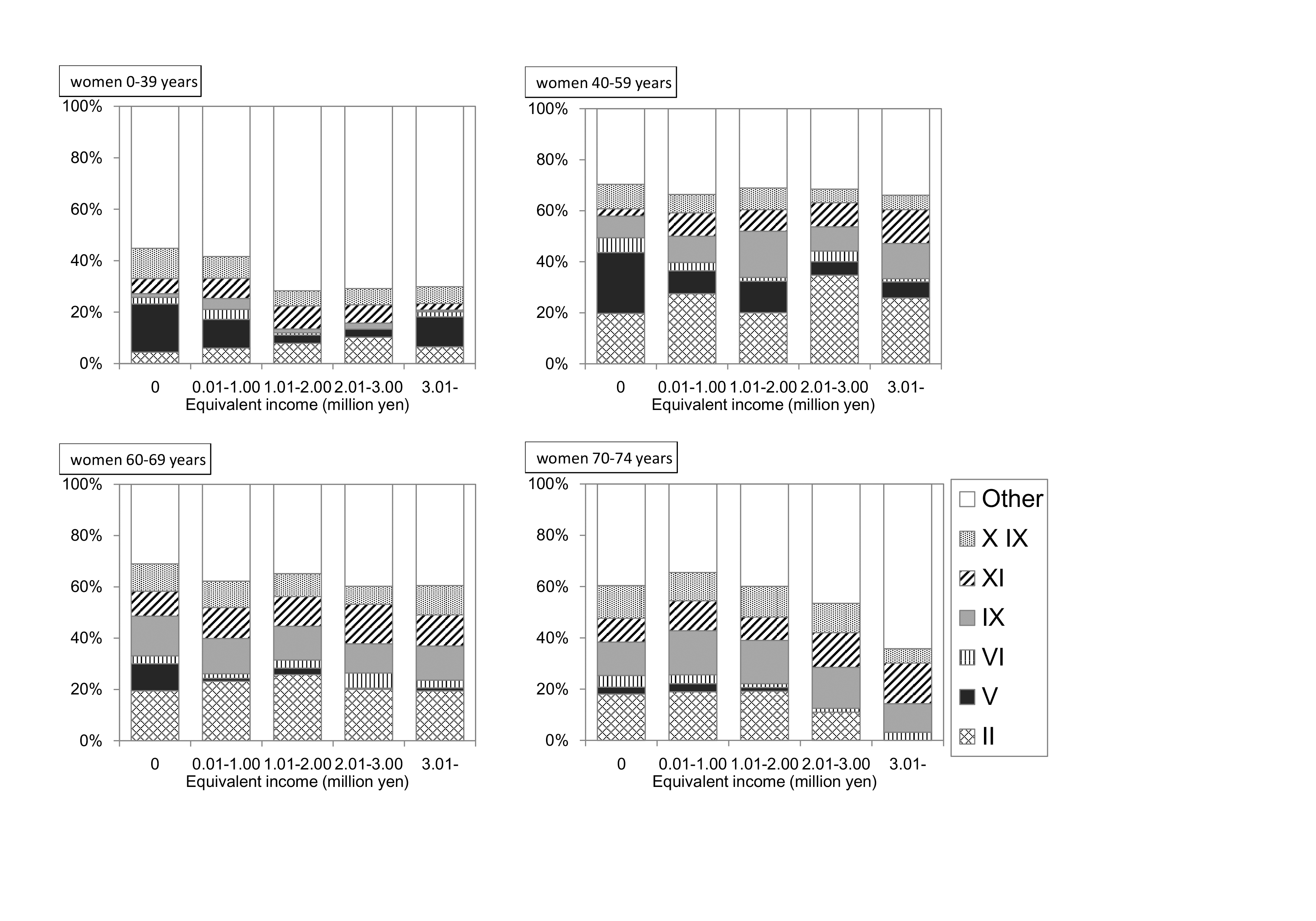

Supplement: S2 Fig — The proportion of claims with each diagnosis to the total number of hospitalization claims is shown. The number of claims in this tabulation was 8,911. II: Neoplasms. V: Mental and behavioral disorders. VI: Disease of the nervous system. IX: Disease of the circulatory system. XI: Disease of the digestive system. XIX: Injury, poisoning and certain other consequences of external causes. (TIF) [file pone.0151690.s002.tif]
