# Supplementary material for: Income Related Inequality of Health Care Access in Japan: A Retrospective Cohort Study
Source: PLoS One. 2016 Mar 15;11(3):e0151690. doi: 10.1371/journal.pone.0151690 (PMC4792389; doi:10.1371/journal.pone.0151690)
Supplement: S1 Table — Abbreviations: CI, confidence interval; QIC, quasi-likelihood under the independence model criterion. aAge is expressed as years. bEquivalent income is expressed as million yen. The number of subjects was 222,259. Binominal distribution and logit link function were defined in this model. (DOCX) [file pone.0151690.s003.docx]

**S1 Table. Coefficients estimated by the generalized estimating equation for the association between equivalent income and utilization of outpatient care services**

|  | Model 1 |  |  | Model 2 |  |  | Model 3 |  |  | Model 4 |  |  | Model 5 |  |  |
| --- | --- | --- | --- | --- | --- | --- | --- | --- | --- | --- | --- | --- | --- | --- | --- |
|  | Coefficients | 95% CI | *P*-value | Coefficients | 95% CI | *P*-value | Coefficients | 95% CI | *P*-value | Coefficients | 95% CI | *P*-value | Coefficients | 95% CI | *P*-value |
| Intercept | 3.33 | 3.26, 3.41 | <0.001 | 3.42 | 3.33, 3.50 | <0.001 | 3.39 | 3.22, 3.56 | <0.001 | 3.49 | 3.31, 3.67 | <0.001 | 3.24 | 2.98, 3.51 | <0.001 |
| Sex |  |  |  |  |  |  |  |  |  |  |  |  |  |  |  |
| Men | -0.58 | -0.60, -0.56 | <0.001 | -0.74 | -0.81, -0.67 | <0.001 | -0.59 | -0.62, -0.57 | <0.001 | -0.73 | -0.81, -0.66 | <0.001 | -0.40 | -0.73, -0.07 | 0.016 |
| Women | reference |  |  | reference |  |  | reference |  |  | reference |  |  | reference |  |  |
| Age^a^ |  |  |  |  |  |  |  |  |  |  |  |  |  |  |  |
| 0-15 | -0.51 | -0.58, 0.45 | <0.001 | -0.50 | -0.56, -0.43 | <0.001 | -0.66 | -0.93, -0.38 | <0.001 | -0.66 | -0.93, -0.39 | <0.001 | -0.85 | -1.23 -0.47 | <0.001 |
| 16-39 | -1.71 | -1.75, -1.67 | <0.001 | -1.69 | -1.74, -1.65 | <0.001 | -1.82 | -2.00, -1.64 | <0.001 | -1.83 | -2.01, -1.65 | <0.001 | -1.35 | -1.63, -1.07 | <0.001 |
| 40-59 | -1.67 | -1.71, -1.62 | <0.001 | -1.65 | -1.69, -1.61 | <0.001 | -1.64 | -1.81, -1.46 | <0.001 | -1.66 | -1.83, -1.48 | <0.001 | -1.39 | -1.67, -1.12 | <0.001 |
| 60-69 | -0.75 | -0.79, -0.70 | <0.001 | -0.73 | -0.77, -0.69 | <0.001 | -0.75 | -0.93, -0.56 | <0.001 | -0.76 | -0.94, -0.57 | <0.001 | -0.77 | -1.06, -0.49 | <0.001 |
| 70-74 | reference |  |  | reference |  |  | reference |  |  | reference |  |  | reference |  |  |
| Income^b^ |  |  |  |  |  |  |  |  |  |  |  |  |  |  |  |
| 0.00 | -0.72 | -0.76, -0.67 | <0.001 | -0.72 | -0.74, -0.65 | <0.001 | -0.88 | -1.06, -0.70 | <0.001 | -0.93 | -1.12, -0.74 | <0.001 | -0.64 | -0.91, -0.36 | <0.001 |
| 0.01-1.00 | -0.41 | -0.45, -0.36 | <0.001 | -0.53 | -0.60, -0.46 | <0.001 | -0.51 | -0.68, -0.33 | <0.001 | -0.64 | -0.83, -0.46 | <0.001 | -0.57 | -0.84, -0.30 | <0.001 |
| 1.01-2.00 | -0.26 | -0.31, -0.22 | <0.001 | -0.43 | -0.50, -0.36 | <0.001 | -0.13 | -0.31, 0.05 | 0.146 | -0.32 | -0.51, -0.13 | 0.001 | -0.24 | -0.52, 0.04 | 0.089 |
| 2.01-3.00 | -0.25 | -0.30, -0.20 | <0.001 | -0.36 | -0.44, -0.27 | <0.001 | -0.16 | -0.37, 0.04 | 0.125 | -0.28 | -0.50, -0.06 | 0.013 | -0.35 | -0.67, -0.02 | 0.036 |
| 3.01- | reference |  |  | reference |  |  | reference |  |  | reference |  |  | reference |  |  |
| Sex*age |  |  |  |  |  |  |  |  |  |  |  |  |  |  |  |
| Men*0-15 |  |  |  |  |  |  |  |  |  |  |  |  | 0.39 | -0.09, 0.88 | 0.109 |
| Men*16-39 |  |  |  |  |  |  |  |  |  |  |  |  | -0.70 | -1.05, -0.34 | <0.001 |
| Men*40-59 |  |  |  |  |  |  |  |  |  |  |  |  | -0.40 | -0.74, -0.04 | 0.030 |
| Men*60-69 |  |  |  |  |  |  |  |  |  |  |  |  | 0.08 | -0.29, 0.45 | 0.660 |
| Sex*income |  |  |  |  |  |  |  |  |  |  |  |  |  |  |  |
| Men*0.00 |  |  |  | -0.01 | -0.09, 0.08 | 0.910 |  |  |  | -0.08 | -0.16, 0.02 | 0.110 | -0.62 | -0.99, -0.26 | <0.001 |
| Men*0.01-1.00 |  |  |  | 0.20 | 0.12, 0.29 | <0.001 |  |  |  | 0.20 | 0.11, 0.28 | <0.001 | 0.12 | -0.23, 0.46 | 0.502 |
| Men*1.01-2.00 |  |  |  | 0.27 | 0.19, 0.36 | <0.001 |  |  |  | 0.27 | 0.18, 0.35 | <0.001 | 0.17 | -0.18, 0.53 | 0.335 |
| Men*2.01-3.00 |  |  |  | 0.17 | 0.07, 0.27 | <0.001 |  |  |  | 0.17 | 0.07, 0.27 | 0.001 | 0.28 | -0.13, 0.68 | 0.184 |
| Age*income |  |  |  |  |  |  |  |  |  |  |  |  |  |  |  |
| 0-15*0.00 |  |  |  |  |  |  | 0.23 | -0.08, 0.53 | 0.145 | 0.33 | 0.02, 0.64 | 0.038 | -0.10 | -0.51, 0.31 | 0.633 |
| 0-15*0.01-1.00 |  |  |  |  |  |  | 0.19 | -0.10, 0.48 | 0.190 | 0.20 | -0.09, 0.49 | 0.185 | 0.20 | -0.20, 0.60 | 0.329 |
| 0-15*1.01-2.00 |  |  |  |  |  |  | -0.02 | -0.31, 0.28 | 0.911 | -0.01 | -0.31, 0.29 | 0.954 | 0.00 | -0.41, 0.40 | 0.982 |
| 0-15*2.01-3.00 |  |  |  |  |  |  | 0.16 | -0.18, 0.51 | 0.357 | 0.17 | -0.18, 0.51 | 0.343 | 0.27 | -0.21, 0.75 | 0.267 |
| 16-39*0.00 |  |  |  |  |  |  | 0.35 | 0.15, 0.54 | <0.001 | 0.44 | 0.24, 0.64 | <0.001 | -0.05 | -0.35, 0.26 | 0.761 |
| 16-39*0.01-1.00 |  |  |  |  |  |  | 0.21 | 0.02, 0.40 | 0.028 | 0.22 | 0.03, 0.41 | 0.021 | 0.11 | -0.19, 0.42 | 0.460 |
| 16-39*1.01-2.00 |  |  |  |  |  |  | -0.13 | -0.32, 0.06 | 0.184 | -0.12 | -0.31, 0.08 | 0.244 | -0.24 | -0.55, 0.07 | 0.124 |
| 16-39*2.01-3.00 |  |  |  |  |  |  | -0.02 | -0.25, 0.20 | 0.835 | -0.01 | -0.24, 0.21 | 0.908 | 0.01 | -0.35, 0.38 | 0.938 |
| 40-59*0.00 |  |  |  |  |  |  | 0.19 | -0.01, 0.38 | 0.058 | 0.30 | 0.10, 0.49 | 0.003 | -0.05 | -0.35, 0.24 | 0.722 |
| 40-59*0.01-1.00 |  |  |  |  |  |  | 0.05 | -0.13, 0.24 | 0.574 | 0.08 | -0.11, 0.27 | 0.404 | 0.03 | -0.27, 0.32 | 0.857 |
| 40-59*1.01-2.00 |  |  |  |  |  |  | -0.27 | -0.46, -0.08 | 0.005 | -0.24 | -0.44, -0.05 | 0.013 | -0.26 | -0.56, 0.04 | 0.090 |
| 40-59*2.01-3.00 |  |  |  |  |  |  | -0.24 | -0.46, -0.01 | 0.038 | -0.22 | -0.44, 0.01 | 0.061 | -0.12 | -0.47, 0.23 | 0.497 |
| 60-69*0.00 |  |  |  |  |  |  | -0.05 | -0.26, 0.15 | 0.594 | 0.00 | -0.21, 0.20 | 0.976 | 0.00 | -0.31, 0.31 | 0.994 |
| 60-69*0.01-1.00 |  |  |  |  |  |  | 0.07 | -0.12, 0.26 | 0.485 | 0.09 | -0.11, 0.28 | 0.378 | 0.19 | -0.11, 0.49 | 0.217 |
| 60-69*1.01-2.00 |  |  |  |  |  |  | -0.08 | -0.27, 0.12 | 0.450 | -0.05 | -0.25, 0.15 | 0.627 | 0.00 | -0.30, 0.31 | 0.979 |
| 60-69*2.01-3.00 |  |  |  |  |  |  | -0.02 | -0.26, 0.21 | 0.844 | -0.08 | -0.24, 0.23 | 0.948 | 0.18 | -0.18, 0.54 | 0.326 |
| Sex*age * income |  |  |  |  |  |  |  |  |  |  |  |  |  |  |  |
| Men*0-16*0.00 |  |  |  |  |  |  |  |  |  |  |  |  | 0.82 | 0.29, 1.37 | 0.003 |
| Men*0-15*0.01-1.00 |  |  |  |  |  |  |  |  |  |  |  |  | -0.01 | -0.53, 0.50 | 0.957 |
| Men*0-15*1.01-2.00 |  |  |  |  |  |  |  |  |  |  |  |  | 0.01 | -0.52, 0.54 | 0.962 |
| Men*0-15*2.01-3.00 |  |  |  |  |  |  |  |  |  |  |  |  | -0.14 | -0.77, 0.49 | 0.659 |
| Men*16-39*0.00 |  |  |  |  |  |  |  |  |  |  |  |  | 0.83 | 0.42, 1.23 | <0.001 |
| Men*16-39*0.01-1.00 |  |  |  |  |  |  |  |  |  |  |  |  | 0.09 | -0.29, 0.47 | 0.651 |
| Men*16-39*1.01-2.00 |  |  |  |  |  |  |  |  |  |  |  |  | 0.15 | -0.24, 0.54 | 0.446 |
| Men*16-39*2.01-300 |  |  |  |  |  |  |  |  |  |  |  |  | -0.06 | -0.51, 0.40 | 0.809 |
| Men*40-59*0.00 |  |  |  |  |  |  |  |  |  |  |  |  | 0.64 | 0.24, 1.03 | 0.002 |
| Men*40-59*0.01-1.00 |  |  |  |  |  |  |  |  |  |  |  |  | 0.03 | -0.35, 0.40 | 0.894 |
| Men*40-59*1.01-2.00 |  |  |  |  |  |  |  |  |  |  |  |  | -0.01 | -0.39, 0.37 | 0.954 |
| Men*40-59*2.01-3.00 |  |  |  |  |  |  |  |  |  |  |  |  | -0.15 | -0.59, 0.29 | 0.505 |
| Men*60-69*0.00 |  |  |  |  |  |  |  |  |  |  |  |  | 0.03 | -0.39, 0.44 | 0.905 |
| Men*60-69*0.01-1.00 |  |  |  |  |  |  |  |  |  |  |  |  | -0.20 | -0.60, 0.19 | 0.306 |
| Men*60-69*1.01-2.00 |  |  |  |  |  |  |  |  |  |  |  |  | -0.10 | -0.50, 0.29 | 0.607 |
| Men*60-69*2.01-3.00 |  |  |  |  |  |  |  |  |  |  |  |  | -0.31 | -0.77, 0.15 | 0.189 |
| Residence area |  |  |  |  |  |  |  |  |  |  |  |  |  |  |  |
| Chuo | -0.06 | -0.11, -0.02 | 0.002 | -0.06 | -0.10, -0.02 | 0.003 | -0.06 | -0.10, -0.02 | 0.003 | -0.06 | -0.10, -0.02 | 0.004 | -0.06 | -0.10, -0.02 | 0.005 |
| Hanamigawa | -0.07 | -0.11, -0.02 | 0.002 | -0.06 | -0.11, -0.02 | 0.003 | -0.06 | -0.11, -0.02 | 0.003 | -0.06 | -0.10, -0.02 | 0.004 | -0.06 | -0.10, -0.02 | 0.005 |
| Inage | -0.04 | -0.08, 0.00 | 0.071 | -0.04 | -0.08, 0.00 | 0.082 | -0.04 | -0.08, 0.00 | 0.065 | -0.04 | -0.08, 0.00 | 0.077 | -0.04 | -0.08, 0.01 | 0.102 |
| Wakaba | -0.06 | -0.10, -0.02 | 0.003 | -0.06 | -0.10, -0.02 | 0.005 | -0.06 | -0.10, -0.02 | 0.004 | -0.06 | -0.10, -0.02 | 0.008 | -0.05 | -0.09, -0.01 | 0.016 |
| Midori | 0.05 | 0.00, 0.10 | 0.032 | 0.05 | 0.01, 0.10 | 0.029 | 0.05 | 0.01, 0.10 | 0.028 | 0.06 | 0.01, 0.10 | 0.024 | 0.06 | 0.01, 0.10 | 0.022 |
| Mihama | reference |  |  | reference |  |  | reference |  |  | reference |  |  | reference |  |  |
| Number of family members |  |  |  |  |  |  |  |  |  |  |  |  |  |  |  |
| 1 or 2 | -0.12 | -0.16, -0.08 | <0.001 | -0.12 | -0.16, -0.07 | <0.001 | -0.14 | -0.19, -0.10 | <0.001 | -0.14 | -0.18, -0.10 | <0.001 | -0.13 | -0.17, -0.09 | <0.001 |
| 3 | -0.04 | -0.09, 0.00 | 0.062 | -0.04 | -0.09, 0.00 | 0.062 | -0.05 | -0.10, -0.01 | 0.022 | -0.06 | -0.10, -0.01 | 0.018 | -0.05 | -0.09, 0.00 | 0.047 |
| 4 or more | reference |  |  | reference |  |  | reference |  |  | reference |  |  | reference |  |  |
| QIC | 197,521 |  |  | 197,436 |  |  | 197,330 |  |  | 197,211 |  |  | 196,692 |  |  |

Abbreviations: CI, confidence interval; QIC, quasi-likelihood under the independence model criterion

^a^Age is expressed as years.

^b^Equivalent income is expressed as million yen.

The number of subjects was 222,259.

Binominal distribution and logit link function were defined in this model.
