# Supplementary material for: Income Related Inequality of Health Care Access in Japan: A Retrospective Cohort Study
Source: PLoS One. 2016 Mar 15;11(3):e0151690. doi: 10.1371/journal.pone.0151690 (PMC4792389; doi:10.1371/journal.pone.0151690)
Supplement: S3 Table — Abbreviations: CI, confidence interval; QIC, quasi-likelihood under the independence model criterion. aAge is expressed as years. bEquivalent income is expressed as million yen. The number of subjects who received outpatient care services in survey year was 180,256. Poisson distribution and log link function were defined in this model. (DOCX) [file pone.0151690.s005.docx]

**S3 Table. Coefficients estimated by the generalized estimating equation for the association between equivalent income and duration of outpatient care**

|  | Model 1 |  |  | Model 2 |  |  | Model 3 |  |  | Model 4 |  |  | Model 5 |  |  |
| --- | --- | --- | --- | --- | --- | --- | --- | --- | --- | --- | --- | --- | --- | --- | --- |
|  | Coefficients | 95% CI | *P*-value | Coefficients | 95% CI | *P*-value | Coefficients | 95% CI | *P*-value | Coefficients | 95% CI | *P*-value | Coefficients | 95% CI | *P*-value |
| Intercept | 2.96 | 2.92, 2.99 | <0.001 | 2.97 | 2.93, 3.00 | <0.001 | 2.98 | 2.94, 3.03 | <0.001 | 2.99 | 2.94, 3.04 | <0.001 | 3.01 | 2.95, 3.07 | <0.001 |
| Sex |  |  |  |  |  |  |  |  |  |  |  |  |  |  |  |
| Men | -0.09 | -0.11, -0.08 | <0.001 | -0.11 | -0.14, -0.07 | <0.001 | -0.10 | -0.11, -0.09 | <0.001 | -0.11 | -0.15, -0.08 | <0.001 | -0.15 | -0.22, -0.08 | <0.001 |
| Women | reference |  |  | reference |  |  | reference |  |  | reference |  |  | reference |  |  |
| Age^a^ |  |  |  |  |  |  |  |  |  |  |  |  |  |  |  |
| 0-15 | -0.66 | -0.68, -0.63 | <0.001 | -0.66 | -0.68, -0.63 | <0.001 | -0.53 | -0.60, -0.46 | <0.001 | -0.53 | -0.61, -0.46 | <0.001 | -0.66 | -0.75, -0.57 | <0.001 |
| 16-39 | -1.08 | -1.10, -1.06 | <0.001 | -1.08 | -1.10, -1.06 | <0.001 | -1.11 | -1.17, -1.05 | <0.001 | -1.11 | -1.17, -1.05 | <0.001 | -1.07 | -1.15, -0.10 | <0.001 |
| 40-59 | -0.63 | -0.65, -0.60 | <0.001 | -0.63 | -0.65, -0.61 | <0.001 | -0.72 | -0.77, -0.66 | <0.001 | -0.72 | -0.77, -0.67 | <0.001 | -0.72 | -0.79, -0.65 | <0.001 |
| 60-69 | -0.29 | -0.30, -0.28 | <0.001 | -0.29 | -0.31, -0.28 | <0.001 | -0.31 | -0.36, -0.27 | <0.001 | -0.31 | -0.36, -0.26 | <0.001 | -0.35 | -0.41, -0.28 | <0.001 |
| 70-74 | reference |  |  | reference |  |  | reference |  |  | reference |  |  | reference |  |  |
| Income^b^ |  |  |  |  |  |  |  |  |  |  |  |  |  |  |  |
| 0.00 | 0.14 | 0.11, 0.16 | <0.001 | 0.12 | 0.09, 0.15 | <0.001 | 0.07 | 0.02, 0.12 | 0.003 | 0.06 | 0.01, 0.11 | 0.014 | 0.06 | 0.00, 0.12 | 0.047 |
| 0.01-1.00 | 0.07 | 0.05, 0.09 | <0.001 | 0.06 | 0.03, 0.09 | <0.001 | 0.05 | 0.00, 0.09 | 0.036 | 0.03 | -0.02, 0.08 | 0.205 | 0.02 | -0.04, 0.08 | 0.459 |
| 1.01-2.00 | 0.04 | 0.02, 0.06 | <0.001 | 0.04 | 0.01, 0.06 | 0.009 | 0.03 | -0.01, 0.07 | 0.158 | 0.02 | -0.02, 0.07 | 0.335 | 0.03 | -0.03, 0.08 | 0.376 |
| 2.01-3.00 | 0.01 | -0.01, 0.04 | 0.403 | 0.03 | 0.00, 0.06 | 0.082 | 0.00 | -0.06, 0.05 | 0.856 | 0.01 | -0.04, 0.07 | 0.644 | -0.01 | -0.08, 0.06 | 0.842 |
| 3.01- | reference |  |  | reference |  |  | reference |  |  | reference |  |  | reference |  |  |
| Sex*age |  |  |  |  |  |  |  |  |  |  |  |  |  |  |  |
| Men*0-15 |  |  |  |  |  |  |  |  |  |  |  |  | 0.26 | 0.14, 0.38 | <0.001 |
| Men*16-39 |  |  |  |  |  |  |  |  |  |  |  |  | -0.09 | -0.20, 0.02 | 0.123 |
| Men*40-59 |  |  |  |  |  |  |  |  |  |  |  |  | -0.01 | -0.12, 0.10 | 0.862 |
| Men*60-69 |  |  |  |  |  |  |  |  |  |  |  |  | 0.09 | -0.01, 0.18 | 0.073 |
| Sex*income |  |  |  |  |  |  |  |  |  |  |  |  |  |  |  |
| Men*0.00 |  |  |  | 0.05 | 0.00, 0.10 | 0.067 |  |  |  | 0.03 | -0.03, 0.08 | 0.334 | -0.05 | -0.16, 0.07 | 0.422 |
| Men*0.01-1.00 |  |  |  | 0.03 | -0.01, 0.07 | 0.201 |  |  |  | 0.03 | -0.08, 0.07 | 0.115 | 0.05 | -0.03, 0.13 | 0.221 |
| Men*1.01-2.00 |  |  |  | 0.01 | -0.03, 0.05 | 0.541 |  |  |  | 0.02 | -0.02, 0.05 | 0.430 | 0.01 | -0.07, 0.09 | 0.776 |
| Men*2.01-3.00 |  |  |  | -0.04 | -0.09, 0.01 | 0.125 |  |  |  | -0.03 | -0.08, 0.02 | 0.203 | 0.01 | -0.09, 0.11 | 0.834 |
| Age*income |  |  |  |  |  |  |  |  |  |  |  |  |  |  |  |
| 0-15*0.00 |  |  |  |  |  |  | -0.25 | -0.34, -0.16 | <0.001 | -0.25 | -0.35, -0.16 | <0.001 | -0.23 | -0.34, -0.11 | <0.001 |
| 0-15*0.01-1.00 |  |  |  |  |  |  | -0.18 | -0.26, -0.10 | <0.001 | -0.18 | -0.26, -0.10 | <0.001 | -0.14 | -0.24, -0.04 | 0.008 |
| 0-15*1.01-2.00 |  |  |  |  |  |  | -0.06 | -0.14, 0.02 | 0.132 | -0.06 | -0.14, 0.02 | 0.135 | -0.08 | -0.18, 0.03 | 0.140 |
| 0-15*2.01-3.00 |  |  |  |  |  |  | -0.06 | -0.16, 0.03 | 0.195 | -0.06 | -0.16, 0.03 | 0.178 | -0.06 | -0.18, 0.07 | 0.365 |
| 16-39*0.00 |  |  |  |  |  |  | 0.07 | -0.01, 0.14 | 0.072 | 0.07 | -0.10, 0.14 | 0.085 | 0.05 | -0.04, 0.15 | 0.274 |
| 16-39*0.01-1.00 |  |  |  |  |  |  | 0.09 | 0.02, 0.16 | 0.011 | 0.09 | 0.02, 0.16 | 0.010 | 0.10 | 0.00, 0.19 | 0.040 |
| 16-39*1.01-2.00 |  |  |  |  |  |  | -0.03 | -0.10, 0.04 | 0.355 | -0.03 | -0.10, 0.04 | 0.364 | -0.03 | -0.12, 0.06 | 0.497 |
| 16-39*2.01-3.00 |  |  |  |  |  |  | -0.02 | -0.10, 0.06 | 0.635 | -0.02 | -0.10, 0.06 | 0.604 | -0.01 | -0.12, 0.09 | 0.803 |
| 40-59*0.00 |  |  |  |  |  |  | 0.24 | 0.17, 0.31 | <0.001 | 0.24 | 0.17, 0.31 | <0.001 | 0.21 | 0.12, 0.30 | <0.001 |
| 40-59*0.01-1.00 |  |  |  |  |  |  | 0.10 | 0.03, 0.16 | 0.003 | 0.10 | 0.04, 0.17 | 0.002 | 0.05 | -0.03, 0.14 | 0.202 |
| 40-59*1.01-2.00 |  |  |  |  |  |  | 0.03 | -0.03, 0.10 | 0.329 | 0.03 | -0.03, 0.10 | 0.305 | 0.04 | -0.05, 0.12 | 0.408 |
| 40-59*2.01-3.00 |  |  |  |  |  |  | 0.01 | -0.07, 0.09 | 0.724 | 0.01 | -0.07, 0.09 | 0.736 | 0.06 | -0.05, 0.16 | 0.299 |
| 60-69*0.00 |  |  |  |  |  |  | 0.05 | -0.01, 0.12 | 0.105 | 0.05 | -0.01, 0.12 | 0.105 | 0.07 | -0.01, 0.14 | 0.094 |
| 60-69*0.01-1.00 |  |  |  |  |  |  | 0.01 | -0.04, 0.07 | 0.656 | 0.02 | -0.04, 0.07 | 0.588 | 0.05 | -0.02, 0.13 | 0.154 |
| 60-69*1.01-2.00 |  |  |  |  |  |  | 0.00 | -0.06, 0.05 | 0.962 | 0.00 | -0.06, 0.05 | 0.992 | 0.00 | -0.07, 0.08 | 0.905 |
| 60-69*2.01-3.00 |  |  |  |  |  |  | 0.03 | -0.04, 0.09 | 0.433 | 0.02 | -0.04, 0.09 | 0.525 | 0.06 | -0.03, 0.15 | 0.173 |
| Sex*age * income |  |  |  |  |  |  |  |  |  |  |  |  |  |  |  |
| Men*0-15*0.00 |  |  |  |  |  |  |  |  |  |  |  |  | 0.02 | -0.15, 0.19 | 0.806 |
| Men*0-15*0.01-1.00 |  |  |  |  |  |  |  |  |  |  |  |  | -0.09 | -0.22, 0.05 | 0.203 |
| Men*0-15*1.01-2.00 |  |  |  |  |  |  |  |  |  |  |  |  | 0.03 | -0.11, 0.16 | 0.708 |
| Men*0-15*2.01-3.00 |  |  |  |  |  |  |  |  |  |  |  |  | -0.02 | -0.18, 0.14 | 0.813 |
| Men*16-39*0.00 |  |  |  |  |  |  |  |  |  |  |  |  | 0.11 | -0.05, 0.27 | 0.187 |
| Men*16-39*0.01-1.00 |  |  |  |  |  |  |  |  |  |  |  |  | -0.02 | -0.16, 0.12 | 0.800 |
| Men*16-39*1.01-2.00 |  |  |  |  |  |  |  |  |  |  |  |  | 0.00 | -0.13, 0.14 | 0.951 |
| Men*16-39*2.01-300 |  |  |  |  |  |  |  |  |  |  |  |  | -0.01 | -0.17, 0.15 | 0.929 |
| Men*40-59*0.00 |  |  |  |  |  |  |  |  |  |  |  |  | 0.15 | -0.01, 0.30 | 0.061 |
| Men*40-59*0.01-1.00 |  |  |  |  |  |  |  |  |  |  |  |  | 0.13 | 0.00, 0.26 | 0.056 |
| Men*40-59*1.01-2.00 |  |  |  |  |  |  |  |  |  |  |  |  | 0.00 | -0.13, 0.13 | 0.977 |
| Men*40-59*2.01-3.00 |  |  |  |  |  |  |  |  |  |  |  |  | -0.08 | -0.24, 0.08 | 0.325 |
| Men*60-69*0.00 |  |  |  |  |  |  |  |  |  |  |  |  | 0.06 | -0.09, 0.21 | 0.434 |
| Men*60-69*0.01-1.00 |  |  |  |  |  |  |  |  |  |  |  |  | -0.09 | -0.19, 0.02 | 0.112 |
| Men*60-69*1.01-2.00 |  |  |  |  |  |  |  |  |  |  |  |  | -0.01 | -0.12, 0.09 | 0.829 |
| Men*60-69*2.01-3.00 |  |  |  |  |  |  |  |  |  |  |  |  | -0.09 | -0.21, 0.04 | 0.184 |
| Residence area |  |  |  |  |  |  |  |  |  |  |  |  |  |  |  |
| Chuo | 0.01 | -0.01, 0.03 | 0.480 | 0.01 | -0.01, 0.03 | 0.497 | 0.01 | -0.01, 0.03 | 0.495 | 0.01 | -0.01, 0.03 | 0.496 | 0.01 | -0.01, 0.03 | 0.486 |
| Hanamigawa | -0.02 | -0.04, 0.00 | 0.112 | -0.02 | -0.04, 0.00 | 0.109 | -0.02 | -0.04, 0.00 | 0.116 | -0.02 | -0.04, 0.00 | 0.116 | -0.02 | -0.04, 0.00 | 0.118 |
| Inage | -0.06 | -0.08, -0.04 | <0.001 | -0.06 | -0.08, -0.04 | <0.001 | -0.06 | -0.08, -0.04 | <0.001 | -0.06 | -0.08, -0.04 | <0.001 | -0.06 | -0.08, -0.04 | <0.001 |
| Wakaba | -0.06 | -0.08, -0.04 | <0.001 | -0.06 | -0.08, -0.04 | <0.001 | -0.06 | -0.08, -0.04 | <0.001 | -0.06 | -0.08, 0.04 | <0.001 | -0.06 | -0.08, -0.04 | <0.001 |
| Midori | 0.07 | 0.05, 0.10 | <0.001 | 0.07 | 0.05, 0.10 | <0.001 | 0.07 | 0.05, 0.10 | <0.001 | 0.07 | 0.05, 0.10 | <0.001 | 0.08 | 0.05, 0.10 | <0.001 |
| Mihama | reference |  |  | reference |  |  | reference |  |  | reference |  |  | reference |  |  |
| Number of family members |  |  |  |  |  |  |  |  |  |  |  |  |  |  |  |
| 1 or 2 | 0.19 | 0.17, 0.22 | <0.001 | 0.19 | 0.17, 0.21 | <0.001 | 0.19 | 0.17, 0.22 | <0.001 | 0.19 | 0.17, 0.22 | <0.001 | 0.19 | 0.17, 0.22 | <0.001 |
| 3 | 0.16 | 0.13, 0.19 | <0.001 | 0.16 | 0.13, 0.19 | <0.001 | 0.16 | 0.14, 0.19 | <0.001 | 0.16 | 0.14, 0.19 | <0.001 | 0.16 | 0.14, 0.19 | <0.001 |
| 4 or more | reference |  |  | reference |  |  | reference |  |  | reference |  |  | reference |  |  |
| QIC | -427,832 |  |  | -428,161 |  |  | -429,339 |  |  | -429,508 |  |  | -429,865 |  |  |

Abbreviations: CI, confidence interval; QIC, quasi-likelihood under the independence model criterion

^a^Age is expressed as years.

^b^Equivalent income is expressed as million yen.

The number of subjects who received outpatient care services in the survey year was 180,256.

Poisson distribution and log link function were defined in this model.
