# Supplementary material for: Income Related Inequality of Health Care Access in Japan: A Retrospective Cohort Study
Source: PLoS One. 2016 Mar 15;11(3):e0151690. doi: 10.1371/journal.pone.0151690 (PMC4792389; doi:10.1371/journal.pone.0151690)
Supplement: S4 Table — Abbreviations: CI, confidence interval; QIC, quasi-likelihood under the independence model criterion. aAge is expressed as years. bEquivalent income is expressed as million yen. The number of subjects who were hospitalized in the survey year was 15,425. Poisson distribution and log link function were defined in this model. (DOCX) [file pone.0151690.s006.docx]

**S4 Table. Coefficients estimated by the generalized estimating equation for the association between equivalent income and duration of hospitalization**

|  | Model 1 |  |  | Model 2 |  |  | Model 3 |  |  | Model 4 |  |  | Model 5 |  |  |
| --- | --- | --- | --- | --- | --- | --- | --- | --- | --- | --- | --- | --- | --- | --- | --- |
|  | Coefficients | 95% CI | *P*-value | Coefficients | 95% CI | *P*-value | Coefficients | 95% CI | *P*-value | Coefficients | 95% CI | *P*-value | Coefficients | 95% CI | *P*-value |
| Intercept | 2.26 | 2.03, 2.48 | <0.001 | 2.36 | 2.09, 2.63 | <0.001 | 2.41 | 2.11, 2.70 | <0.001 | 2.50 | 2.13, 2.86 | <0.001 | 2.60 | 2.14, 3.06 | <0.001 |
| Sex |  |  |  |  |  |  |  |  |  |  |  |  |  |  |  |
| Men | 0.10 | 0.03, 0.17 | 0.003 | -0.03 | -0.29, 0.23 | 0.815 | 0.08 | 0.01, 0.15 | 0.022 | -0.05 | -0.33, 0.22 | 0.706 | -0.23 | -0.74, 0.27 | 0.359 |
| Women | reference |  |  | reference |  |  | reference |  |  | reference |  |  | reference |  |  |
| Age^a^ |  |  |  |  |  |  |  |  |  |  |  |  |  |  |  |
| 0-39 | -0.02 | -0.15, 0.12 | 0.818 | -0.04 | -0.18, 0.10 | 0.558 | 0.06 | -0.37, 0.49 | 0.791 | 0.03 | -0.42, 0.48 | 0.890 | -0.37 | -1.00, 0.26 | 0.247 |
| 40-59 | 0.56 | 0.46, 0.66 | <0.001 | 0.53 | 0.43, 0.64 | <0.001 | -0.06 | -0.46, 0.34 | 0.779 | -0.07 | -0.48, 0.34 | 0.733 | -0.27 | -0.83, 0.29 | 0.346 |
| 60-69 | 0.24 | 0.17, 0.32 | <0.001 | 0.23 | 0.15, 0.30 | <0.001 | 0.18 | -0.14, 0.50 | 0.267 | 0.17 | -0.15, 0.49 | 0.292 | 0.16 | -0.37, 0.69 | 0.551 |
| 70-74 | reference |  |  | reference |  |  | reference |  |  | reference |  |  | reference |  |  |
| Equivalent income^b^ |  |  |  |  |  |  |  |  |  |  |  |  |  |  |  |
| 0.00 | 1.07 | 0.92, 1.22 | <0.001 | 0.93 | 0.72, 1.15 | <0.001 | 0.70 | 0.42, 0.98 | <0.001 | 0.59 | 0.24, 0.94 | 0.001 | 0.44 | -0.02, 0.89 | 0.058 |
| 0.01-1.00 | 0.38 | 0.23, 0.52 | <0.001 | 0.26 | 0.04, 0.47 | 0.019 | 0.28 | 0.03, 0.53 | 0.029 | 0.13 | -0.21, 0.47 | 0.447 | 0.12 | -0.32, 0.57 | 0.589 |
| 1.01-2.00 | 0.14 | -0.01, 0.29 | 0.059 | 0.20 | -0.02, 0.42 | 0.075 | 0.09 | -0.16, 0.35 | 0.475 | 0.15 | -0.20, 0.50 | 0.394 | 0.04 | -0.41, 0.49 | 0.857 |
| 2.01-3.00 | -0.07 | -0.25, 0.11 | 0.451 | 0.01 | -0.26, 0.28 | 0.941 | -0.04 | -0.33, 0.26 | 0.816 | 0.06 | -0.33, 0.45 | 0.760 | -0.16 | -0.68, 0.35 | 0.534 |
| 3.01- | reference |  |  | reference |  |  | reference |  |  | reference |  |  | reference |  |  |
| Sex*age |  |  |  |  |  |  |  |  |  |  |  |  |  |  |  |
| Men*0-39 |  |  |  |  |  |  |  |  |  |  |  |  | 0.77 | -0.07, 1.61 | 0.074 |
| Men*40-59 |  |  |  |  |  |  |  |  |  |  |  |  | 0.34 | -0.45, 1.12 | 0.404 |
| Men*60-69 |  |  |  |  |  |  |  |  |  |  |  |  | -0.01 | -0.67, 0.65 | 0.977 |
| Sex*equivalent income |  |  |  |  |  |  |  |  |  |  |  |  |  |  |  |
| Men*0.00 | - |  |  | 0.27 | -0.02, 0.56 | 0.070 |  |  |  | 0.25 | -0.05, 0.55 | 0.106 | 0.55 | -0.04, 1.14 | 0.07 |
| Men*0.01-1.00 |  |  |  | 0.20 | -0.08, 0.48 | 0.169 |  |  |  | 0.22 | -0.07, 0.52 | 0.141 | 0.25 | -0.28, 0.78 | 0.358 |
| Men*1.01-2.00 |  |  |  | -0.10 | -0.39, 0.18 | 0.478 |  |  |  | -0.09 | -0.39, 0.21 | 0.562 | 0.08 | -0.46, 0.62 | 0.768 |
| Men*2.01-3.00 |  |  |  | -0.13 | -0.48, 0.22 | 0.466 |  |  |  | -0.14 | -0.50, 0.22 | 0.452 | 0.21 | -0.41, 0.83 | 0.503 |
| Age*equivalent income |  |  |  |  |  |  |  |  |  |  |  |  |  |  |  |
| 0-39*0.00 |  |  |  |  |  |  | 0.06 | -0.43, 0.56 | 0.803 | 0.06 | -0.45, 0.57 | 0.814 | 0.36 | -0.36, 1.08 | 0.327 |
| 0-39*0.01-1.00 |  |  |  |  |  |  | -0.11 | -0.59, 0.37 | 0.648 | -0.06 | -0.57, 0.43 | 0.794 | 0.05 | -0.66, 0.75 | 0.893 |
| 0-39*1.01-2.00 |  |  |  |  |  |  | -0.06 | -0.56, 0.44 | 0.812 | -0.09 | -0.62, 0.43 | 0.735 | 0.03 | -0.69, 0.75 | 0.937 |
| 0-39*2.01-3.00 |  |  |  |  |  |  | -0.33 | -0.90, 0.23 | 0.242 | -0.37 | -0.95, 0.21 | 0.213 | 0.05 | -0.75, 0.85 | 0.904 |
| 40-59*0.00 |  |  |  |  |  |  | 0.85 | 0.41, 1.29 | <0.001 | 0.82 | 0.37, 1.27 | <0.001 | 1.24 | 0.63, 1.84 | <0.001 |
| 40-59*0.01-1.00 |  |  |  |  |  |  | 0.64 | 0.21, 1.08 | 0.004 | 0.67 | 0.23, 1.12 | 0.003 | 0.73 | 0.11, 1.34 | 0.021 |
| 40-59*1.01-2.00 |  |  |  |  |  |  | 0.61 | 0.14, 1.07 | 0.010 | 0.60 | 0.12, 1.07 | 0.014 | 0.82 | 0.16, 1.47 | 0.014 |
| 40-59*2.01-3.00 |  |  |  |  |  |  | 0.28 | -0.27, 0.82 | 0.325 | 0.27 | -0.28, 0.82 | 0.341 | 0.66 | -0.15, 1.47 | 0.112 |
| 60-69*0.00 |  |  |  |  |  |  | 0.44 | 0.08, 0.81 | 0.016 | 0.42 | 0.06, 0.79 | 0.022 | 0.39 | -0.17, 0.96 | 0.175 |
| 60-69*0.01-1.00 |  |  |  |  |  |  | -0.06 | -0.40, 0.28 | 0.729 | -0.04 | -0.39, 0.30 | 0.800 | -0.16 | -0.72, 0.40 | 0.580 |
| 60-69*1.01-2.00 |  |  |  |  |  |  | -0.17 | -0.52, 0.17 | 0.323 | -0.19 | -0.53, 0.16 | 0.295 | -0.12 | -0.69, 0.45 | 0.686 |
| 60-69*2.01-3.00 |  |  |  |  |  |  | -0.17 | -0.58, 0.24 | 0.418 | -0.19 | -0.60, 0.22 | 0.370 | 0.01 | -0.66, 0.68 | 0.980 |
| Sex*age *equivalent income |  |  |  |  |  |  |  |  |  |  |  |  |  |  |  |
| Men*0-39*0.00 |  |  |  |  |  |  |  |  |  |  |  |  | -0.63 | -1.61, 0.35 | 0.209 |
| Men*0-39*0.01-1.00 |  |  |  |  |  |  |  |  |  |  |  |  | -0.28 | -1.22, 0.65 | 0.554 |
| Men*0-39*1.01-2.00 |  |  |  |  |  |  |  |  |  |  |  |  | -0.12 | -1.10, 0.86 | 0.815 |
| Men*0-39*2.01-3.00 |  |  |  |  |  |  |  |  |  |  |  |  | -0.71 | -1.82, 0.39 | 0.207 |
| Men*40-59*0.00 |  |  |  |  |  |  |  |  |  |  |  |  | -0.75 | -1.62, 0.13 | 0.094 |
| Men*40-59*0.01-1.00 |  |  |  |  |  |  |  |  |  |  |  |  | -0.10 | -0.96, 0.76 | 0.813 |
| Men*40-59*1.01-2.00 |  |  |  |  |  |  |  |  |  |  |  |  | -0.38 | -1.29, 0.54 | 0.422 |
| Men*40-59*2.01-3.00 |  |  |  |  |  |  |  |  |  |  |  |  | -0.66 | -1.74, 0.43 | 0.236 |
| Men*60-69*0.00 |  |  |  |  |  |  |  |  |  |  |  |  | 0.04 | -0.72, 0.80 | 0.922 |
| Men*60-69*0.01-1.00 |  |  |  |  |  |  |  |  |  |  |  |  | 0.20 | -0.51, 0.91 | 0.580 |
| Men*60-69*1.01-2.00 |  |  |  |  |  |  |  |  |  |  |  |  | -0.11 | -0.82, 0.61 | 0.773 |
| Men*60-69*2.01-3.00 |  |  |  |  |  |  |  |  |  |  |  |  | -0.30 | -1.16, 0.55 | 0.488 |
| Residence area |  |  |  |  |  |  |  |  |  |  |  |  |  |  |  |
| Chuo | 0.15 | 0.04, 0.27 | 0.010 | 0.15 | 0.03, 0.26 | 0.011 | 0.15 | 0.04, 0.27 | 0.009 | 0.15 | 0.04, 0.27 | 0.01 | 0.15 | 0.03, 0.26 | 0.012 |
| Hanamigawa | 0.09 | -0.02, 0.21 | 0.118 | 0.09 | -0.03, 0.21 | 0.123 | 0.10 | -0.02, 0.21 | 0.098 | 0.10 | -0.02, 0.21 | 0.104 | 0.10 | -0.02, 0.21 | 0.099 |
| Inage | 0.02 | -0.10, 0.15 | 0.705 | 0.02 | -0.10, 0.15 | 0.705 | 0.03 | -0.10, 0.15 | 0.685 | 0.03 | -0.10, 0.15 | 0.693 | 0.02 | -0.10, 0.14 | 0.745 |
| Wakaba | 0.08 | -0.03, 0.20 | 0.159 | 0.08 | -0.04, 0.20 | 0.179 | 0.09 | -0.03, 0.21 | 0.135 | 0.08 | -0.03, 0.20 | 0.155 | 0.09 | -0.03, 0.21 | 0.140 |
| Midori | 0.19 | 0.05, 0.32 | 0.006 | 0.18 | 0.05, 0.31 | 0.006 | 0.19 | 0.06, 0.32 | 0.004 | 0.19 | 0.06, 0.32 | 0.005 | 0.19 | 0.06, 0.32 | 0.005 |
| Mihama | reference |  |  | reference |  |  | reference |  |  | reference |  |  | reference |  |  |
| Number of family members |  |  |  |  |  |  |  |  |  |  |  |  |  |  |  |
| 1 or 2 | 0.48 | 0.31, 0.64 | <0.001 | 0.47 | 0.31, 0.63 | <0.001 | 0.48 | 0.32, 0.64 | <0.001 | 0.48 | 0.31, 0.64 | <0.001 | 0.49 | 0.33, 0.65 | <0.001 |
| 3 | 0.46 | 0.29, 0.64 | <0.001 | 0.46 | 0.28, 0.63 | <0.001 | 0.46 | 0.29, 0.64 | <0.001 | 0.46 | 0.28, 0.63 | <0.001 | 0.47 | 0.29, 0.64 | <0.001 |
| 4 or more | reference |  |  | reference |  |  | reference |  |  | reference |  |  | reference |  |  |
| QIC | -20,804 |  |  | -20,914 |  |  | -21,073 |  |  | -21,174 |  |  | -21,361 |  |  |

Abbreviations: CI, confidence interval; QIC, quasi-likelihood under the independence model criterion

^a^Age is expressed as years.

^b^Equivalent income is expressed as million yen.

The number of subjects who were hospitalized in the survey year was 15,425.

Poisson distribution and log link function were defined in this model.
